# Rapid Assessment of CRISPR Transfection Efficiency and Enrichment of CRISPR Induced Mutations Using a Dual-Fluorescent Stable Reporter System

**DOI:** 10.3389/fgeed.2022.854866

**Published:** 2022-03-21

**Authors:** Karim E. Shalaby, Mustapha Aouida, Vijay Gupta, Simona S. Ghanem, Omar M. A. El-Agnaf

**Affiliations:** ^1^ Biological and Biomedical Sciences Division, College of Health and Life Sciences, Hamad Bin Khalifa University, Doha, Qatar; ^2^ Neurological Disorders Research Center, Qatar Biomedical Research Institute (QBRI), Hamad Bin Khalifa University (HBKU), Qatar Foundation, Doha, Qatar

**Keywords:** stable reporter, rapid cell-based assay, CRISPR-Cas9, uptake, enrichment

## Abstract

The nuclease activity of the CRISPR-Cas9 system relies on the delivery of a CRISPR-associated protein 9 (Cas9) and a single guide RNA (sgRNA) against the target gene. CRISPR components are typically delivered to cells as either a Cas9/sgRNA ribonucleoprotein (RNP) complex or a plasmid encoding a Cas9 protein along with a sequence-specific sgRNA. Multiple transfection reagents are known to deliver CRISPR-Cas9 components, and delivery vectors are being developed for different purposes by several groups. Here, we repurposed a dual-fluorescence (RFP-GFP-GFP) reporter system to quantify the uptake level of the functional CRISPR-Cas9 components into cells and compare the efficiency of CRISPR delivery vectors. Using this system, we developed a novel and rapid cell-based microplate reader assay that makes possible real-time, rapid, and high throughput quantification of CRISPR nuclease activity. Cells stably expressing this dual-fluorescent reporter construct facilitated a direct quantification of the level of the internalized and functional CRISPR-Cas9 molecules into the cells without the need of co-transfecting fluorescently labeled reporter molecules. Additionally, targeting a reporter gene integrated into the genome recapitulates endogenous gene targeting. Thus, this reporter could be used to optimize various transfection conditions of CRISPR components, to evaluate and compare the efficiency of transfection agents, and to enrich cells containing desired CRISPR-induced mutations.

## Introduction

Genome editing using the Clustered Regularly Interspaced Short Pallindromic Repeats (CRISPR)-Cas9 system entails the delivery of Cas9 and gRNA into the cells in the form of a ribonucleoprotein (RNP) composed of a functional Cas9/gRNA complex or a plasmid encoding the two components (Shalaby et al., 2020). In both cases, it is essential that a Cas9/sgRNA RNP complex reaches the cytosol and gains access to the nucleus to perform gene editing at specific sequences guided by sgRNA. For this purpose, several delivery vectors and transfection reagents have been developed (Lostalé-Seijo et al., 2017; Suresh et al., 2017; Chaverra-Rodriguez et al., 2018; Givens et al., 2018; Wilbie et al., 2019; Xu et al., 2019; Shalaby et al., 2020; Wei et al., 2020; Yip, 2020). Biological barriers in the way of such tools such as delivery to the target cells, degradation by serum or cytosolic nucleases, not enough cellular uptake or inefficient endosomal escape are in the way of achieving desirable levels of delivery and gene editing (Givens et al., 2018), (Lino et al., 2018), (Belting et al., 2005). Generally, techniques such as immunostaining and fluorescence microscopy are used along with fluorescently labeled molecules to monitor and quantify the levels of uptake of delivered CRISPR-Cas9 cargo complexes (Rouet et al., 2018; Chung et al., 2019; Jain et al., 2019). These methods offer the direct visualization of the uptake of CRISPR-Cas9 cargo complexes into cells. However, they do not show the level of functionally active CRISPR molecules that have successfully reached the nucleus to perform gene editing.

Various reporters that can detect CRISPR/Cas9 nuclease activity in cells have been developed for different purposes (Kim et al., 2011; Ramakrishna et al., 2014a; Ren et al., 2015; Zhou et al., 2016; Wen et al., 2017; Eki et al., 2020). Such reporters are most commonly employed since NHEJ is the dominant repair pathway cells rely on to repair double-stranded breaks (DSB) (Sansbury et al., 2019). On the other hand, SRIRACCHA and CDDR reporters are able to detect Homology-Directed Repair events (Eki et al., 2020), (Wen et al., 2017). CDDR is additionally a reporter that can discriminate between high-fidelity and error-prone NHEJ, which enables the quantification of DNA repair outcomes and the identification of DNA repair factors (Eki et al., 2020) Furthermore, some reporters, such as C-Check, are based on Single-Stranded Annealing (SSA) repair pathway for detecting nuclease activity (Ren et al., 2015), (Zhou et al., 2016). Overall, these reporters are relatively complex, requiring the co-transfection of multiple elements to function, as we further elaborate and compare in the discussion section.

The reporter system employed here is an RFP-GFP based reporter system that utilizes a reporter construct containing a cloning site for inserting Cas9 target sequences between *RFP* and *GFP* genes. It has been developed for the detection and the enrichment of cells containing CRISPR-Cas9 induced mutations (Suresh et al., 2017), (Ramakrishna et al., 2014a), (Kim et al., 2011), (Ramakrishna et al., 2014b), (Kim et al., 2013). Although this dual-reporter system and similar reporters can detect Cas9 nuclease activity, they are usually co-transfected with the CRISPR-Cas9 cargo complexes and are used episomally. Thus, they cannot be employed to quantify the total level of Cas9/sgRNA uptake within a transfected cell population. Additionally, the requirement for co-transfection of a reporter plasmid and CRISPR-Cas9 cargo complexes may hinder the process of CRISPR-Cas9 delivery or induce undesired cytotoxicity, leading to inconsistent or inaccurate results. We describe here the development of stable reporter cells expressing a reporter system based on fluorescent RFP-GFP to accurately measure functional CRISPR-Cas9 cargo uptake to assess the efficiency of transfection agents. Unlike in the episomal system, targeting a reporter gene stably integrated into the genome of the cells recapitulates endogenous gene targeting. We describe here the development of a rapid microplate reader approach to quantifying CRISPR nuclease activity using the reporter system. Furthermore, we show that the system can be used to efficiently enrich cells containing Cas9 mutations for the generation of knock-out cell lines.

## Results

### The Strategy of Developing a Stably Transfected Reporter Cell Line to Detect CRISPR-Cas9 Nuclease Activity

**FIGURE 1:**
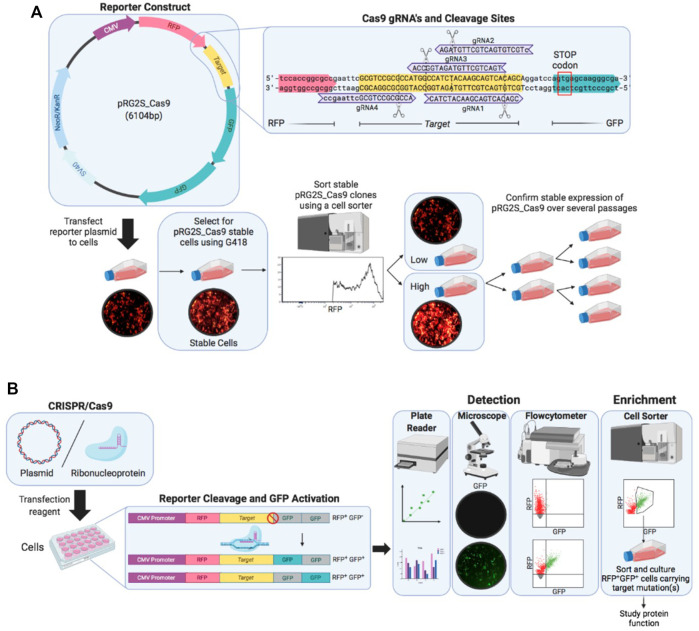
Overview of the stable reporter system for detection of CRISPR-Cas9 induced mutations in human cells. **(A)** Fluorescent reporter construct map highlighting reporter-guide RNAs (gRNAs) sequences (purple) and binding sites at the Cas9 target sequence (yellow) engineered and cloned using *BstXI* and *BamHI* restriction enzymes between *RFP* (red) and *GFP* (green) genes. A stable reporter cell line is generated by selection of stable transformants of the fluorescent reporter construct using G418 antibiotic and sorting stable transformants using a cell sorter. **(B)** Illustration of the working mechanism of the fluorescent reporter. The *RFP* gene (red), upstream of Cas9 target sequence (yellow), is constitutively expressed under CMV promoter (purple), while two *GFP* genes (grey) are cloned out of frame (+1 and +2) downstream of the Cas9 target sequence and they are expressed only if a suitable frameshift mutation occurs through error-prone non-homologous end-joining (NHEJ) DNA repair after double-stranded breaks are introduced by Cas9 nuclease at the target sequence. A frameshift mutation circumvents the stop-codon preventing GFP expression causes either of the *GFP* genes in-frame to be expressed (green). Cells expressing the reporter construct are treated with CRISPR-Cas9 and gRNA expression plasmid or ribonucleoprotein complex and nuclease activity is detected under a fluorescence microscope and using flow cytometry.

To detect CRISPR-Cas9 nuclease activity in cells, we designed a dual-fluorescence reporter system (pRG2S_Cas9) that facilitates selection of cells expressing the reporter system and cells that have undergone gene editing (Figure 1A). The system constitutively expresses mRFP regardless of any nuclease activity as a marker to enrich cells expressing the reporter construct using flowcytometry. mRFP is followed by a spacer between the mRFP and a stop codon which comprises a Cas9-target sequence where we cloned Cas9:gRNA target sites. The second fluorophore, eGFP, cloned twice out-of-frame (frames +1 and +2) downstream of the stop codon (Figure 1A), allows for the selection of nuclease active cells as previously described (Ramakrishna et al., 2014a). In the presence of functionally active Cas9/sgRNA complexes in the cells delivered as plasmid DNA or RNP, the Cas9 nuclease guided by a sgRNA creates double-stranded breaks in the Cas9-target sequence triggering error-prone Non-homologous End-Joining (NHEJ) DNA repair. This pathway often leads to insertions and deletions (indels), which bypass the stop codon and result in either of the eGFP genes being in-frame and permanently activate eGFP expression in cells (Figure 1B). Thus, the percentage of RFP^+^GFP^+^ cells represents the percentage of cells that have undergone gene editing. This system allows for rapid and effective flow cytometry, microscopy, and microplate reader based detection of nuclease-active cells based on double-positive fluorescence (Figure 1B).

### Assessment of Genome Editing Activity Using the Engineered Stable Reporter Cell Line

**FIGURE 2:**
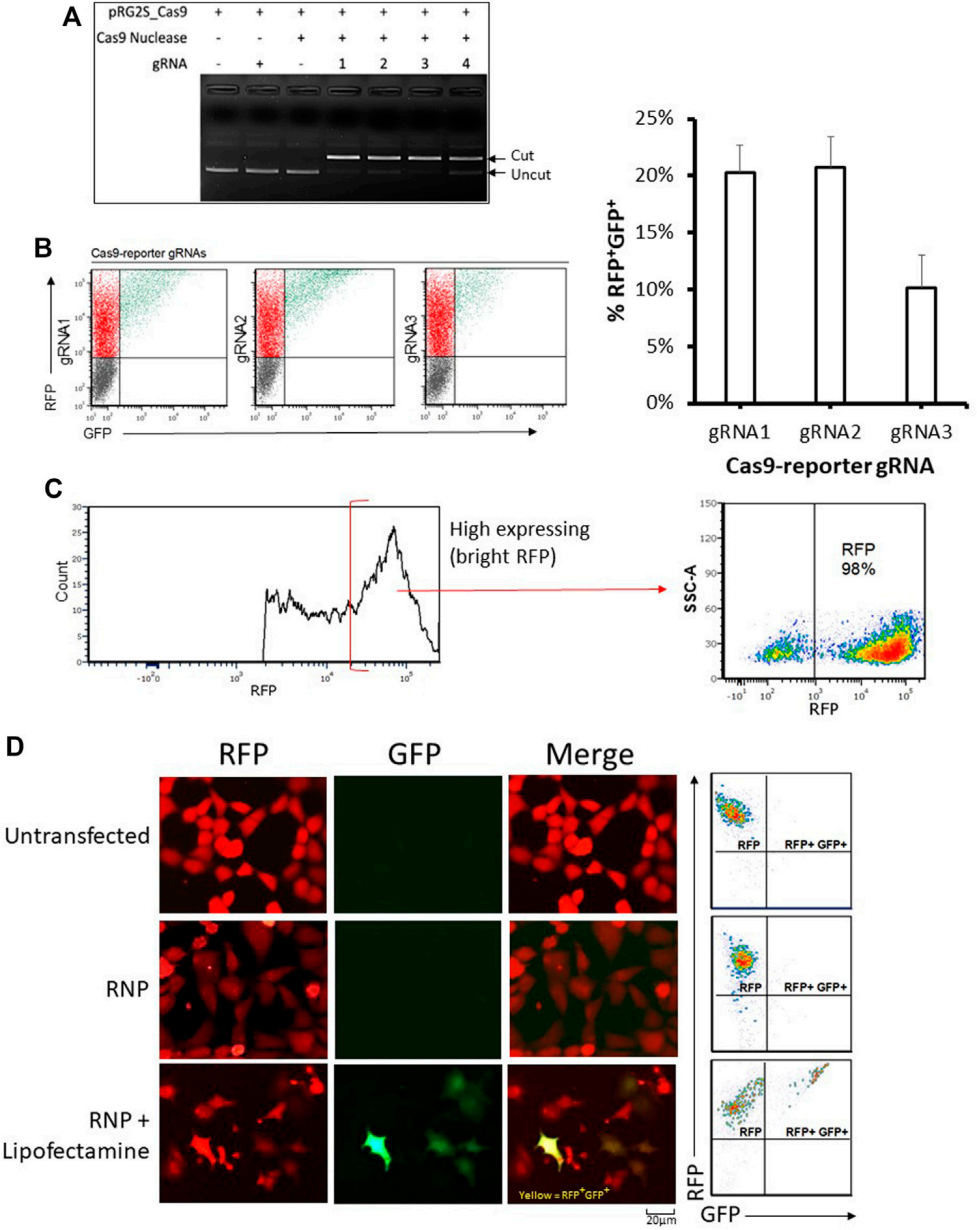
Analysis of genome editing using the stable reporter system. **(A)** *In-vitro* cleavage assay. The four reporter-gRNAs targeting the reporter construct’s Cas9 target sequence are incubated separately with Cas9 nuclease and the reporter plasmid for 1 h at 37°C and then the reactions are subjected to agarose gel electrophoresis to confirm the gRNAs’ ability to induce double-stranded breaks within the target sequence. **(B)** Cellular reporter assay flow-cytometry dot plots. Flow-cytometric analysis of HEK293 cells co-transfected with the reporter plasmid, a Cas9-expression plasmid and DNA fragments encoding each of the four reporter-gRNAs under a U6 promoter, using FuGENE. Quantification of nuclease activity is shown on the right. Results are expressed as mean ± standard error of the mean: *n* = 5 for reporter-gRNA1 and gRNA2; and *n* = 4 for gRNA3. **(C)** A highly stable population of fluorescent reporter cells (∼98% RFP) expressing the reporter construct were sorted using a cell sorter. **(D)** Examples of flow cytometry-based analysis and fluorescence microscopy-based detectionof stable reporter HEK293 cells cultured for 72 h after transfection with Cas9/reporter-gRNA2 ribonucleoprotein using Lipofectamine CRISPRMAX. Scale bar = 20 µm.

We designed gRNAs towards the inserted Cas9 targeting sequence (Figure 1A) and confirmed their functionality *in vitro* (Figure 2A). We then compared their efficiency in HEK293 cells co-transfected with the reporter plasmid, a CRISPR-Cas9 expression plasmid, and either gRNA expression fragments (Figure 2B). The highest nuclease activity was attributed to target reporter-gRNA2 based on the resultant percentage of GFP expressing cells (Figure 2B).

Having determined that the dual reporter construct can be used to detect Cas9 nuclease activity, it was stably incorporated into HEK293 cells that were sorted into low and high expressing populations (Figure 2C and Supplementary Figure S1A). As expected, stable reporter HEK293 cells showed a high expression of mRFP, and only showed expression of eGFP after transfection with plasmids expressing Cas9 and targeting gRNAs (Figures 2C,D and Supplementary Figure S1C). Cas9 target reporter-gRNA2 was again found to be the most efficient in stable reporter cells co-transfected with a CRISPR-Cas9 expression plasmid, and either of the four gRNA expression fragments (Supplementary Figure S1B). The gRNA with the highest efficiency, Cas9 target reporter-gRNA2, was thus selected for further experiments based on the highest GFP expression in cells as measured by flow cytometry (Supplementary Figure S1B). gRNA2 was cloned into the CRISPR-Cas9 expression plasmid, then used as the single plasmid delivery cargo for subsequent experiments. For ribonucleoprotein (RNP) based assays, a Cas9 nuclease complexed with a synthetic gRNA harboring the sequence of Cas9 target reporter-gRNA2 was used.

### Development of a New Rapid Microplate Reader Based Approach to Quantify CRISPR Nuclease Activity

**FIGURE 3:**
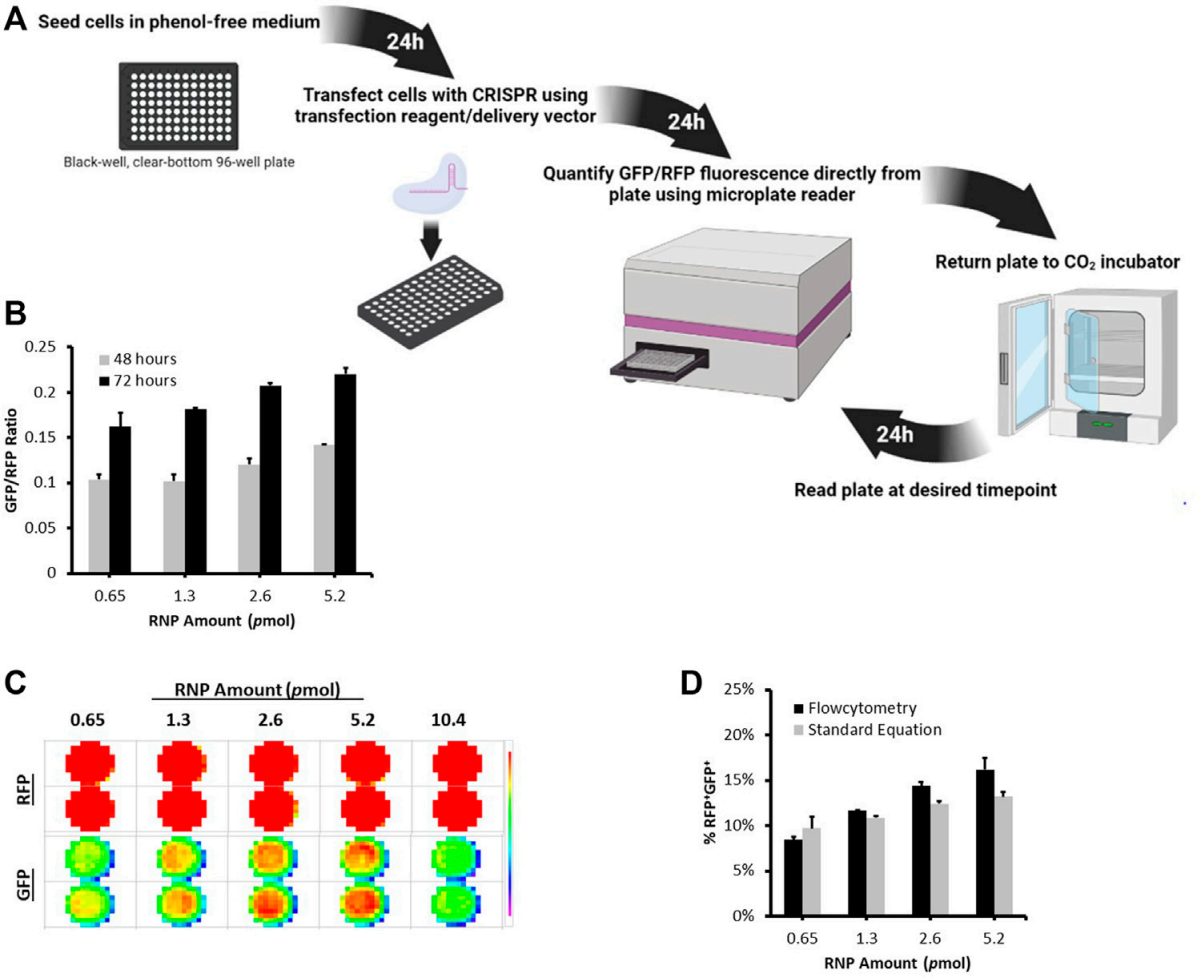
Microplate Reader Approach to Quantifying CRISPR Nuclease Activity Using the Stable Reporter System. **(A)** A schematic illustration of the timeline workflow of the Microplate Reader Assay. Stable Reporter HEK293 cells were seeded at 10,000 per well in a black-well, clear-bottom, 96-well plate. The next day, cells were transfected with different amounts of Cas9/gRNA2 RNP using Lipofectamine CRISPRMAX. **(B)** The plate was scanned for RFP and GFP signal using FLUOstar^®^ Omega microplate at 48 and 72 h post transfection. **(C)** Fluorescence intensity Matrix scan output. **(D)** At 72 h, cells were also analyzed using flowcytometry. The graph shows a comparison between the percentage of RFP^+^GFP^+^ counted using flowctomtery and RFP^+^GFP^+^ values obtained from microplate reader using the formula: %RFP^+^GFP^+^ = (3 (GFP/RFP)/5) * 100. Results are expressed as mean ± standard error of the mean of *n* = 3.

To enably high throughput evaluation of CRISPR delivery efficiency and nuclease activity, we developed a microplate reader based protocol to directly and rapidly quantify CRISPR nuclease activity in cells using the dual-fluorescent stable reporter system (Figure 3, Supplementary Figure S2). We utilized the highly sensitive FLUOstar^®^ Omega multichromatic microplate reader that enables low signal detection levels. We seeded RFP^+^GFP^+^ cells at defined numbers and initial optimization results indicated that the FLUOstar^®^ Omega microplate reader was able to detect a distinct GFP signal at all cell numbers (Supplementary Figure S2A). Results showed a linear relationship between GFP/RFP fluorescence intensity ratio and seeded RFP^+^GFP^+^ cell numbers (Supplementary Figure S2B). This relationship was confirmed by counting RFP^+^GFP^+^ cells using flowcytometry (Supplementary Figure S2C. We were thus able to generate a formula from which the percentage of nuclease active RFP^+^GFP^+^ cells can be determined from the microplate reader’s GFP/RFP fluorescence intensity output (Supplementary Figure S2D).

To demonstrate the applicability of this approach, we transfected stable reporter HEK293 cells with different amounts of Cas9/gRNA2 RNP using Lipofectamine CRISPRMAX and measured nuclease activity at 48 and 72 h using the microplate reader (Figures 3B,C). As expected, different levels of GFP/RFP signals were detected at each RNP amount and increased over time (Figure 3B). Interestingly, the percentages of RFP^+^GFP^+^ cells obtained using flowcytometry were highly similar to the values we calculated using the formula generated above (Figure 3D), further verifying the microplate reader approach as an efficient method for measuring the levels of CRISPR nuclease activity using the dual-fluorescent stable reporter system.

### Reporter-Based Enrichment of Cells Containing CRISPR-Induced Mutations

**FIGURE 4:**
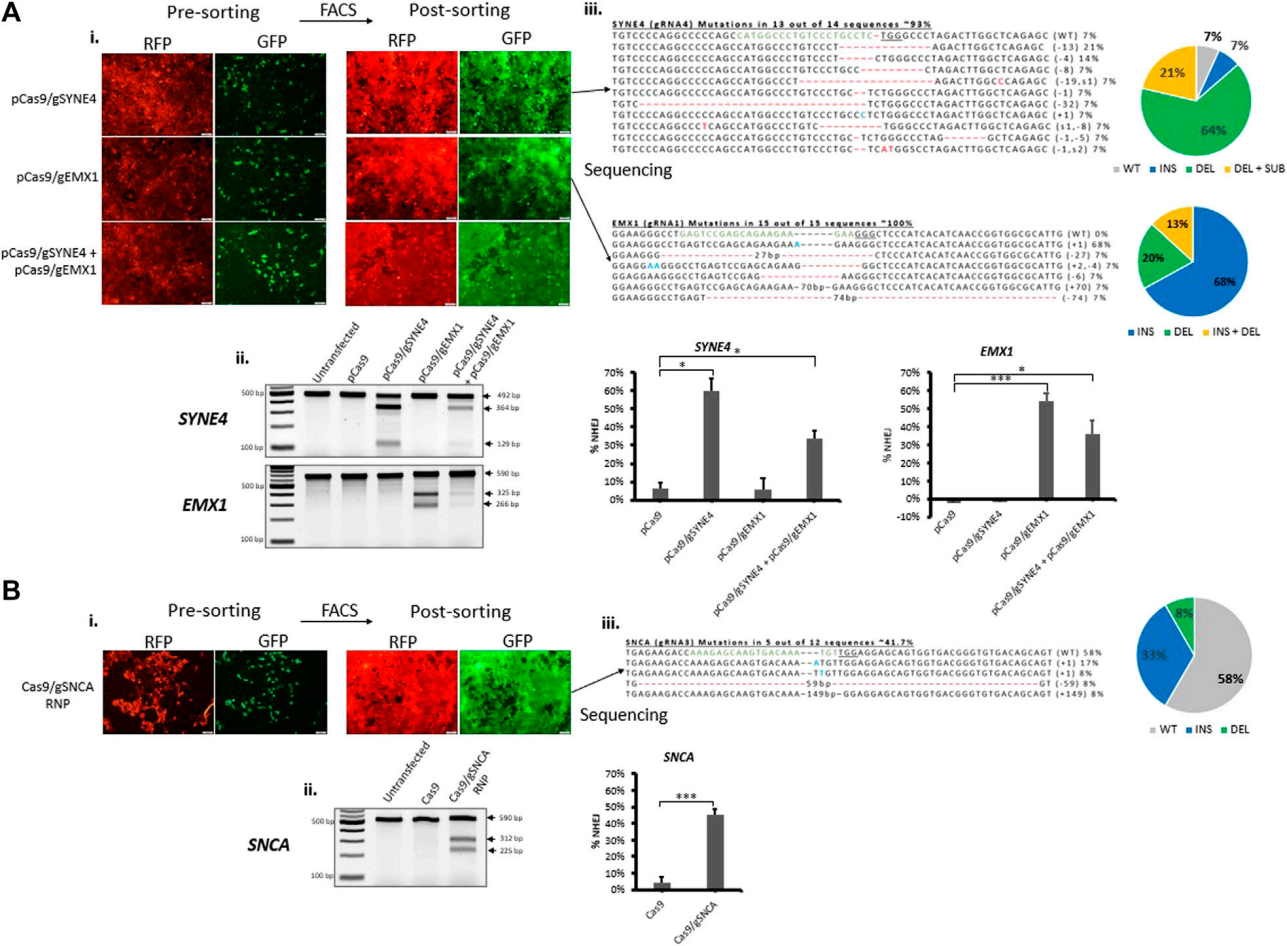
Fluorescence-assisted enrichment of HEK293 cells containing CRISPR-induced mutations. Fluorescent reporter HEK293 cells were transfected with plasmid **(A)** or Ribonucleoprotein (RNP) **(B)** targeting endogenous genes. **(Ai,Bi)** Microscopy images of pre- and post-sorted cells. **(Aii,Bii)** T7 Endonuclease I assay carried out on genomic DNA extracted from post-sorted cells showing indels have been formed in cells treated either with plasmid or RNP targeting endogenous genes. Quantification of indels in T7E1 is shown on the right. Values were background corrected from Untransfected group. Results are expressed as mean ± standard error of the mean of *n* = 3 from T7E1 assay carried out on genomic DNA of targeted cells. There was a significant difference in frequency of indels (*, *p* < 0.05; **, *p* < 0.01; ***, *p* < 0.001) between groups treated with Cas9 alone or Cas9 and gRNA targeting an endogenous gene, as analysed by Student’s *t*-test. **(Aiii,Biii)** Sanger’s sequencing of clones revealing CRISPR-induced indels and mutations. gRNA binding site (green) in the wildtype (WT) sequence is shown, the PAM sequence is underlined, dashes indicate deletions (−), nucleotides colored in blue indicate additions (+), and nucleotide colored in red indicate substitutions (s). Mutation frequencies are calculated as the number of clones carrying a mutation divided by the total number of clones sequenced.

In order to further establish our fluorescent stable reporter system as a means for the enrichment of CRISPR-induced mutations in endogenous genes, we transfected stable fluorescent reporter HEK293 cells with CRISPR plasmid or RNP carrying gRNAs targeting the reporter Cas9-target sequence (Cas9-reporter gRNA2) and endogenous genes simultaneously. As a proof-of-concept, we chose to target *SYNE4*, a gene that encodes nuclear membrane protein nesprin-4 and is associated with hearing loss (Horn et al., 2013), *EMX1* associated with neurodevelopmental and infertility disorders (Zhang et al., 2019), (Kim et al., 2010), and *SNCA* associated with neurodegenerative Parkinson’s Disease (Spillantini et al., 1997), (Bonini and BI, 2005). The percentage of RFP^+^GFP^+^ cells obtained in RNP (Cas9/g*SNCA*) targeted cells (∼16%) (Supplementary Figure S3A) was similar to the percentage we obtained above (Figure 3D). After gene targeting, we sorted pureRFP^+^GFP^+^ cells using fluorescence-assisted cell sorting (Figures 4Ai,Bi). Analysis of indels in RFP^+^GFP^+^ using T7 Endonuclease I (T7EI) assay (Figures 4Aii,Bii revealed significant disruption of endogenous genes in GFP expressing cells using plasmid (targeting *SYNE4* gene: 60%; targeting *EMX1* gene: 56%; targeting *SYNE4* and *EMX1* genes simultaneously: 33 and 36%, respectively) (Figure 4Aii) or RNP (*SNCA* gene: 42%) (Figure 4Bii). The formation of indels in RFP^+^GFP^+^ cells was further confirmed by Sanger’s sequencing of PCR clones (Figures 4Aiii,Biii) and the frequency of indels was analysed by Tracking of Indels by Decomposition (TIDE) assay (Supplementary Figure S4).

**FIGURE 5:**
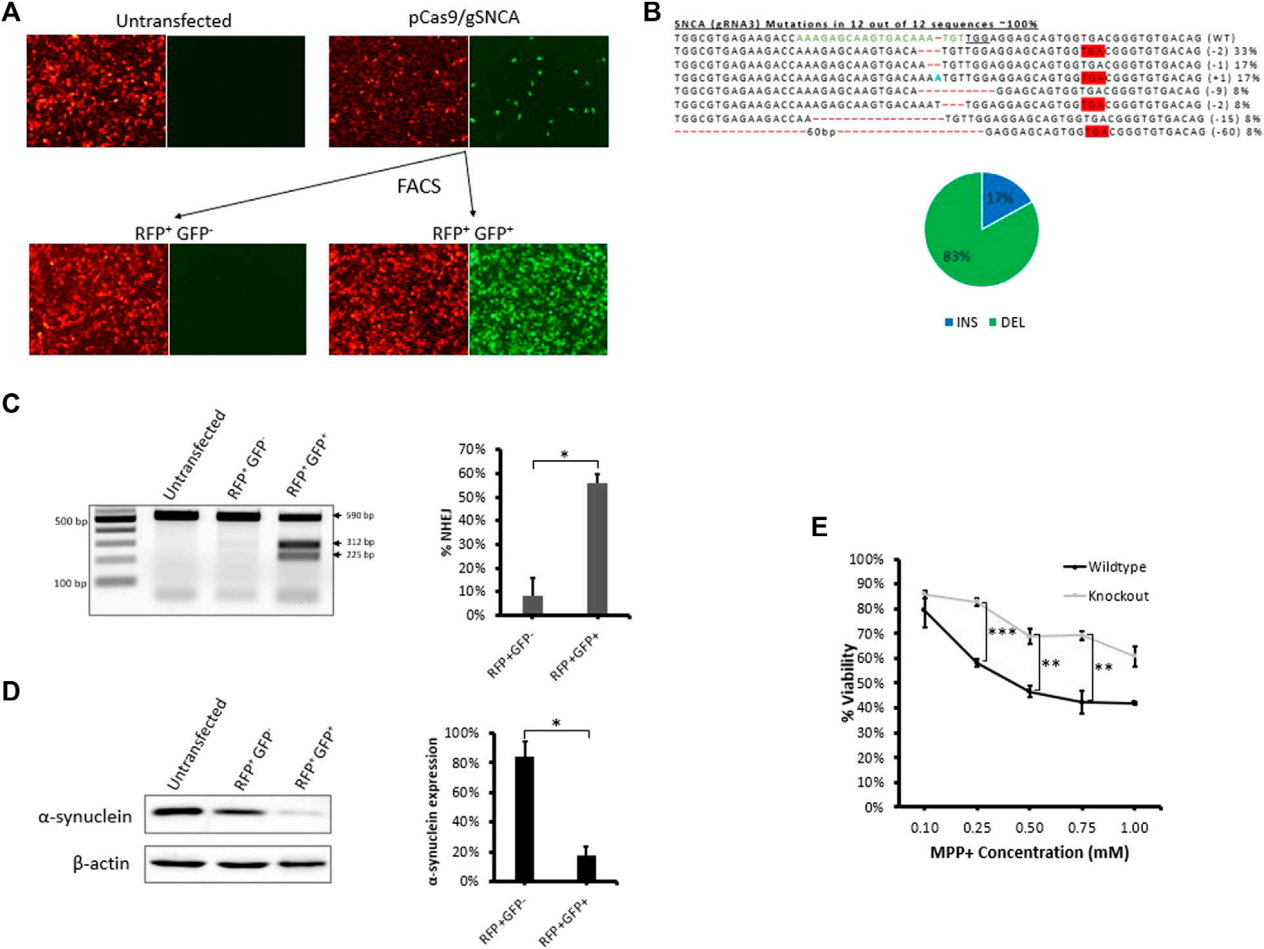
Fluorescence assisted enrichment of knock-out SH-SY5Y cells. **(A)** Microscopy images of pre- and post-sorted fluorescent reporter SH-SY5Y cells transfected with plasmid targeting *SNCA* gene. **(B)** Sanger’s sequencing of clones revealing CRISPR-induced indels and mutations. gRNA binding site (green) in the wildtype (WT) sequence is shown, the PAM sequence is underlined, dashes indicate deletions (-), nucleotides colored in blue indicate additions (+), and nucleotide colored in red indicate substitutions (s). Mutation frequencies (shown on the right) are calculated as the number of clones carrying a mutation divided by the total number of clones sequenced. Mutation frequencies are illustrated in the pie chart: a total of 17% of the mutations lead to insertions, 33% lead to deletions, and 67% lead to the formation of a premature stop codon. **(C)** T7E1 assay carried out on genomic DNA extracted from post-sorted RFP^+^GFP- and RFP^+^GFP^+^ cells confirming the formation of indels in the target region. Quantification of indels in T7E1 is shown on the right. Values were background corrected from Untransfected group. Results are expressed as mean ± standard error of the mean of *n* = 3 from T7E1 assay carried out on genomic DNA of targeted cells. There was a significant difference in frequency of indels (*, *p* < 0.05) between RFP^+^GFP^−^ and RFP^+^GFP^+^ groups, as analyzed by Student’s *t*-test. **(D)** Western blot evaluation of endogenous α-synuclein protein expression in post-sorted RFP^+^GFP^−^ and RFP^+^GFP^+^ cells. Quantification of protein expression in western blot is shown on the right. Results are expressed as mean ± standard error of the mean of *n* = 3 blots carried out on cell lysates of the same cells. There was a significant difference in protein expression (*, *p* < 0.05) between RFP^+^GFP^−^ and RFP^+^GFP^+^ groups, as analyzed by Student’s *t*-test. **(E)** Cytotoxicity MTT Assay comparing cellular viability of SNCA wildtype reporter SH-SY5Y cells and RFP^+^GFP^+^ SNCA knock-out cells exposed to 1, 2.5, and 5 mM MPP^+^ for 24 h. Results are expressed as mean ± standard error of the mean of *n* = 3. There was a significant difference in cellular viability (**, *p* < 0.01; ***, *p* < 0.001) between SNCA wildtype reporter SH-SY5Y cells and RFP^+^GFP^+^ SNCA knock-out cells, as analysed by Student’s *t*-test.

To demonstrate the applicability of our fluorescent reporter system to other applications and cell types, we employed the same strategy to knockout a protein-coding gene in neuronal SH-SY5Y cells (Figure 5). We chose to target *SNCA*, a gene abundantly expressed in the brain and associated with Parkinson’s disease (Spillantini et al., 1997), (Bonini and BI, 2005). After evaluating gRNAs targeting exon 4 of *SNCA*, we identified *SNCA*-gRNA3 as the most efficient gRNA for reducing the expression of endogenous SNCA protein in SH-SY5Y cells (Supplementary Figure S5). We then transfected stable reporter SH-SY5Y cells with CRISPR targeting the reporter sequence (Cas9-reporter gRNA2) and *SNCA* gene using *SNCA*-gRNA3, simultaneously, and sorted the treated cell pool into RFP^+^GFP^−^ and RFP^+^GFP^+^ cells (Figure 5A and Supplementary Figure S3B) and analyzed sorted cells using T7EI assay and western blot (Figures 5C,D and Supplementary Figure S3C). We found significant levels of gene disruption in RFP^+^GFP^+^ cells (56%) compared with RFP^+^GFP^−^ cells (8%) as shown by T7EI assay (Figure 5C). Similarly, RFP^+^GFP^+^ stable reporter SH-SY5Y cells exhibited a significant diminishment of SNCA protein expression (18% SNCA expression) compared with RFP^+^GFP^−^ (84% SNCA expression) relative to untransfected group as shown by western blot (Figure 5D). This diminished expression is in agreement with Sanger’s sequencing data of PCR clones revealing the formation of premature stop codons in 67% of sequenced clones, possibly knocking out protein expression *via* the process of non-sense mediated decay (Popp and Maquat, 2016) (Figure 5B). Furthermore, sequencing analysis of RNP treated cells revealed the formation of deletions at the target site in 73% of PCR clones (Supplementary Figure S3D).

It is well reported that MPP^+^, the toxic metabolite of the neurotoxin MPTP, induces cell death in neuronal cells expressing SNCA (Dauer et al., 2002; Fountaine et al., 2011; Javed et al., 2016). To evaluate a functional role for SNCA, we tested response of RFP^+^GFP^+^ SNCA knock-out cells to MPP^+^ and detected a significant reduction in MPP^+^-mediated cytotoxicity compared with the wildtype reporter SH-SY5Y cells (Figure 5E). Thus, SNCA is important for mediating the response to the neurotoxin MPTP and knocking out SNCA expression protects neuronal cells from MPP^+^-induced cytotoxicity. These results validate that our fluorescent stable reporter system can be used to enrich cells carrying mutations in multiple endogenous genes and suggest it may facilitate the generation of knockout cell lines useful for downstream applications such as studying protein function.

## Discussion

By repurposing a dual fluorescent reporter previously described (Suresh et al., 2017), (Ramakrishna et al., 2014a), (Kim et al., 2011), (Ramakrishna et al., 2014b), (Kim et al., 2013), we demonstrate here how to utilize this system for real-time and rapid detection of nuclease activity and enrichment of cells containing CRISPR-induced mutations. Cells stably expressing this system can be similarly employed to enrich cells using methods such as magnetic separation or antibody selection (Suresh et al., 2017), (Ramakrishna et al., 2014a), (Kim et al., 2011), (Ramakrishna et al., 2014b), (Kim et al., 2013). Such strategy relies on the supposition that nucleases that edit the reporter target sequence also edit the endogenous gene target at a high probability. In comparison to other reporters, we found that this reporter’s simple design and mechanism was very fitting for optimizing CRISPR transfection conditions, comparing the delivery efficiency of CRISPR delivery vectors, and for the enrichment of cells containing one or more targeted endogenous mutations for the generation of knock-out cell lines. For example, there is no requirement to re-design the reporter with a new target sequence for each genomic target which is required in other reporter systems such as the SRIRACCHA and C-Check reporters (Wen et al., 2017), (Zhou et al., 2016). This allows for a one step generation of stable reporter cells that can serve a general purpose for the experiments described here. The SRIRACCHA GFP reporter design also requires the co-transfection of a reporter plasmid, a donor plasmid that promotes HDR repair, and a RFP expression plasmid for normalization, in addition to Cas9 and gRNA(s) which would substantially limit their use in the context of optimizing transfection conditions without interference from external factors (Wen et al., 2017). In the reporter system used here, there is no requirement for transfection of an additional gRNA for the reporter to function, such as the case with the CDDR reporter, which extends the potential to include gRNA(s) to target endogenous genes simultaneously (Eki et al., 2020). Additionally, “off” reporter systems, such as CDDR, would be limited by the time required for fluorescence expression to completely diminish and thus is not suitable to monitor nuclease activity over time (Eki et al., 2020). The present reporter is an “on” reporter where nuclease activity results in the immediate onset of fluorescence expression. Furthermore, “off” reporter cells with more than one integrated construct may not be sensitive in reporting nuclease activity unless all the integrated constructs have been successfully edited (Eki et al., 2020), unlike with an “on” system where editing at one integrated locus is enough to report nuclease activity. The reporters described are probably designed towards characterizing DNA repair factors (Eki et al., 2020) or characterizing DNA repair pathways, such as SSA, high-fidelity, and non-mutagenic NHEJ repair (Eki et al., 2020), (Zhou et al., 2016). However, we find that the reporter used here is appropriate for the purpose of the study which is simply detecting nuclease activity via the error prone NHEJ repair which leads to gene disruption and/or knock-outs and is likely the primary pathway employed by mammalian cells to repair DSBs (Sansbury et al., 2019). Moreover, the presence of two GFP sequences out-of-frame downstream of the target sequence allows for the detection of +1 and +2 frameshifts presenting an advantage over reporters capable of only detecting mutations leading to a single frame. In such reporters, it would only be possible to detect a third of frameshifts because the downstream reporter exists only on one frame, making it impossible to detect +1 or +3 frameshifts that would not result to GFP expression.

Cells stably expressing this dual fluorescent reporter can be an effective means for optimizing transfection conditions of novelCRISPR delivery vectors, and enrichment of cells containing multiple CRISPR-induced mutations. Given that virtually all transfected cells are reporters, they represent a more sensitive system for the accurate measurement of the total level of uptake of functional CRISPR molecules within a cell population.

Various factors such as transfection medium, cell seeding density, incubation time, or dosage can interfere with a delivery vectors’ transfection efficiency. For example, transfection in a serum-free and antibiotics-free medium may correspond to more efficient delivery with lower levels of anionic serum proteins or antibiotics present, which may interfere with complex formation between cationic liposomes and CRISPR-encoding DNA or RNP. Serum has also been reported to have a negative effect on transfection efficiency by various other mechanisms (Delteil et al., 2000). Additionally, liposome-based reagents increase cell permeability leading to antibiotics being internalized into cells, resulting in toxicity and decreased delivery efficiency. Furthermore, reduced levels of uptake of CRISPR molecules per cell are concurrent with higher seeding densities. As such, it is preferable to perform a gene-editing experiment at the lowest possible cell seeding density while avoiding toxicity. Moreover, their is known that a shorter on-set of nuclease activity is expected using RNP due to the less intracellular processing required for performing genome editing, compared with plasmid that must undergo transcription and translation before nuclease active RNP complexes are formed (Shalaby et al., 2020). Hence, shorter incubation times are required using RNP compared with plasmid because RNP levels in cells decline following 24 h after transfection due to cellular degradation (Schubert et al., 2021). Transfecting higher amounts of CRISPR cargo is more prone to the higher efficiency of internalization and gene editing. However, it is preferrable to adopt the lowest dose possible for CRISPR cargo that would achieve adequate amounts of gene editing to reduce possible toxicity and off-target effects endured at higher doses, reducing overall gene editing efficiency (Wang et al., 2016).

Achieving efficient delivery to the cells of interest has been one of the biggest challenges facing CRISPR application to the clinic so far. As such, a great deal of ongoing research is focused on developing and optimizing novel delivery vectors (Shalaby et al., 2020). We claim that our reporter system is useful for effectively measuring and optimizing the delivery of CRISPR plasmid or RNP using delivery vectors. Essentially, cells stably expressing the fluorescent reporter can directly quantify functional CRISPR uptake in cells without the need for co-transfection of reporters or fluorescently labeled molecules that may interfere with the treatment procedure leading to inconsistent or inaccurate results. This helps in assessing the efficiency of novel delivery vectors with minimal interference from external factors. Since only two thirds of frameshifts (+1 and +2) are detectable with GFP expression, the dual-fluorescent reporter system has a theoretical maximum percentage of GFP expression activation of 66%. Although this percentage may vary according to the context of the targeted genomic site, examination of Cas9-induced indels in over 1,000 genomic sites revealed that ∼80% of indels lead to a frameshift (Chakrabarti et al., 2019). These points may be worth considering during analysis of the delivery efficiency of delivery vectors using this system.

The microplate reader approach we developed here enables accurate high throughput evaluation of transfection efficiency and real-time detection of CRISPR nuclease activity using the stable reporter system (Figure 3 and Supplementary Figure S2). We showed that the FLUOstar^®^ Omega microplate reader can be used to detect nuclease activity at high sensitivity down to an initial seeding density of 1,000 RFP^+^GFP^+^ cells (Supplementary Figure S2A). Furthermore, the fluorescence intensity output showed a linear relationship with data obtained using flowcytometry (Supplementary Figure S2C). Using the protocol described here, we were able to accurately compare nuclease activity of different amounts of RNP transfected into stable HEK293 cells over time. Compared with conventional fluorescence microscopy or flowcytometry, the microplate reader approach allows for accurate and direct detection of CRISPR nuclease activity from the same plate over time without disturbing the cells. Although flowcytomtery is a gold standard quantitative method, the microplate reader provides high throughput, real-time, and quantitative information about CRISPR nuclease activity.

Considering that the reporter gene is integrated in the genome, this reporter system also partially recapitulates endogenous gene targeting and thus can be a means for optimizing CRISPR transfection conditions for targeting endogenous genes simply using a microplate reader, without the need for time-consuming downstream analyses. We showed that this system could also be used to efficiently enrich cells containing CRISPR-induced mutations using fluorescence-assisted cell sorting (Figure 4). We also showed that the system can be extended to other cell types of interest and that it represents a reliable approach to the generation of knock-out cell lines (Figure 5). It has been reported that T7EI assay is not sensitive when indel frequencies are above 30% (Liesche et al., 2016), (Sentmanat et al., 2018). We thus carried out TIDE analysis for the sequencing data for a more accurate quantification of indel frequencies (Supplementary Figure S4; see Supplementary Data for sequences). Interestingly, TIDE analysis showed values that were highly similar to indel frequencies based on PCR clones and total cut efficiencies that were close to T7 assay quantifications (Figures 4, 5, and Supplementary Figure S4). (Liesche et al., 2016), (Sentmanat et al., 2018) (Liesche et al., 2016), (Sentmanat et al., 2018)We could further demonstrate that the system can be employed to study protein function such as SNCA as we showed in the SNCA knock-out cells generated (Figure 5E). Future studies are warranted to implement the stable dual-fluorescent reporter system in other cell types. A lot of potential lies in generating induced pluripotent stem cells expressing this reporter system to study gene function in various set ups.

Genome editing using CRISPR is indeed beneficial in various applications. We demonstrated here that cells stably expressing the dual-fluorescent reporter system can be used to assess transfection conditions, enrich CRISPR-induced mutations, and generate knock-out cell lines that may be useful for a broad range of applications.

## Methods

### gRNAs

gRNAs were designed using Benchling’s online gRNA design tool (see Supplementary Data for sequences) and were synthesized as tracrRNA and crRNA and purchased from Integrated DNA Technologies (IDT Coralville, IA, United States) or as sgRNA (Synthego, CA, United States) or embedded in gRNA expression fragments under a U6 promoter (IDT Inc.). gRNAs were designed using benchling’s online gRNA design tool and purchased from Synthego, and gSNCA4 was purchased from IDT Inc.

### Plasmids and Cloning

To construct the pCas9/gRNA2 plasmid, gRNA2 from U6-pRG2S-Cas9-gRNA2 fragment was cloned into the gRNA scaffold of a Cas9 expression plasmid, pX330-U6-Chimeric_BB-CBh-hSpCas9, a gift from Feng Zhang (Addgene plasmid #42230) by double digestion using *BbsI* purchased from New England Biolabs (NEB, MA 01938, United States). One Shot DH5-α competent *E. coli* cells (Invitrogen, Thermo Fisher Scientific, MA 02451, United States) were transformed with the ligation reaction and plated onto LB agar plates containing 100 μg/ml of Ampicillin (Thermo Fisher Scientific, MA, United States).

pCas9/gSYNE4, pCas9/gEMX1 and pCas9/gSNCA3 plasmids were constructed by adding the gRNA sequence into the Cas9 expression vector using overlapping oligos. Once annealed, the oligos produce a fragment containing the gRNA sequence flanked by *BbsI* overhangs and are cloned by ligation into a Cas9 expression vector linearized by *BbsI*. One Shot DH5-α competent *E. coli* cells were transformed with the ligation reaction and plated onto LB agar plates containing 100 μg/ml of Ampicillin (Thermo Fisher Scientific).

To construct the reporter plasmids (pMRS_Cas9: Catalog #TGEN_dRRR1; pRG2S_Cas9: Catalog#TGEN_dRRM1, ToolGen, Seoul, South Korea), we designed a DNA sequence containing multiple CRISPR-Cas9 gRNA binding sites flanked by *BstXI* and *BamHI* cloning sites. The sequence harboring the Cas9 gRNA bindings sites was synthesized by IDT and purchased as a pIDT-plasmid. The targeting sequence was sub-cloned from pIDT into a the reporter plasmids between the mRFP and EGFP fluorescent reporter genes by double digestion using *BstXI* and *BamHI* (NEB). One Shot DH5-α competent *E. coli* cells were transformed with the ligation reaction and plated onto LB agar plates containing 50 μg/ml of Kanamycin (Thermo Fisher Scientific). Positive clones were verified by Sanger’s sequencing using pRG2S forward and reverse primers, synthesized by IDT Inc. (see Supplementary Data for sequences).

### *In-vitro* Cleavage Assay

The Ribonucleoprotein (RNP) complex used in the vitro study was prepared in similar manner described by Saifaldeen et al. (Saifaldeen et al., 2021). Briefly, a final concentration of 50 nM Cas9 nuclease was added to a NEB Buffered solution containing 300 ng pRG2S_Cas9 plasmid and 50 ng gRNA, topped up to 20 μl filter sterilized distilled deionized nuclease-free water. The reaction was allowed to take place at 37°C for 1 h. Results were analyzed on an Ethidium Bromide-stained 0.8% agarose gel using a UV transilluminator (ChemiDoc™ Imaging System, Biorad, CA, United States).

### Cell Culture and Stable Cell Line Preparation

Human Embryonic Kidney cells (HEK293) were maintained in high glucose Dulbecco’s modified Eagle’s medium (DMEM, high glucose, Catalog# Gibco™11965092, Thermo Fisher Scientific) supplemented with 10% foetal bovine serum (Catalog# Gibco™10082147, Thermo Fisher Scientific) 1% penicillin and streptomycin (Catalog# Gibco™15070063, Thermo Fisher Scientific). Neuronal SH-SY5Y cells were maintained in Dulbecco’s Modified Eagle Medium/Nutrient Mixture F-​12 (DMEM/F12 1:1, Catalog# Cytiva HyClone™SH30272.02, Thermo Fisher Scientific) supplemented with 20 mM HEPES buffer (Catalog# Gibco™ 15630080, Thermo Fisher Scientific)), 10% FBS, and 1% penicillin and streptomycin. Cells were grown in an incubator at 37°C in 5% CO_2_ and 95% humidity.

To generate a stable reporter cell line, cells were transfected with 10 μg *BsaI*-linearized pRG2S_Cas9 plasmid (Figure 1A) using FuGENE (Catalog# E2311, Promega, WI, United States) (3 μl/μg). After 3 days, Geniticin G418 Sulphate (Catalog# Gibco™ 0131035, Thermo Fisher Scientific) was added (500 μg/ml for HEK293, 300 μg/ml for SH-SY5Y cells) to select stable cells over a period of 2 weeks. A high (∼98% RFP) and low (∼72% RFP) expressing stable poopulations was sorted using a FACS sorter (BD FacsAria™ II Cell Sorter, BD Biosciences, CA, United States). Fluorescence microscopy was used to confirm RFP expression over several passages.

### Transfection

Choosing the best performing gRNA: HEK293 cells were seeded at 8 × 10^4^ cells per well in 500 ul complete DMEM on a 24-well plate (Catalog# Nunc™ 142475, Thermo Fisher Scientific). After 24 h, cells were co-transfected with 100 ng pMRS_Cas9 reporter plasmid, 150 ng U6-gRNA fragment of different gRNAs, and 500 ng pX330-U6-Chimeric_BB-CBh-hSpCas9 Cas9 expression plasmid using FuGENE (3 μl/μg following manufacturer’s instructions) and incubated for an additional 72 h. At the day of analysis, cells were detached using TrypLE (Catalog# Gibco™12605010, Thermo Fisher Scientific) and collected in DMEM; GFP expression and fluorescence intensity was evaluated using a flow cytometer (LSRFortessa, BD Biosciences).

Comparing gRNA activity in stable cells: HEK293 cells were seeded at 15,000 cells per well in 100 μl complete DMEM on a 96-well plate (Catalog# Nunc™167008, Thermo Fisher Scientific). The next day, cells were co-transfected with 30 ng U6-gRNA fragment of different gRNAs, and 100 ng pX330-U6-Chimeric_BB-CBh-hSpCas9 Cas9 expression plasmid using FuGENE (3 μl/μg). Cells were analyzed after 72 h using flowcytometry.

Microplate reader assay: stable reporter HEK293 cells were seeded at 10,000 per well in 100 µl phenol-free Opti-MEM the day before transfection. Wildtype, non-fluorescent HEK293 cells were seeded at the same density for correction. Cells were then transfected with different amounts of RNP as indicated using Lipofectamine CRISPRMAX (Catalog# CMAX00001, Thermo Fisher Scientific) following the manufacturer’s protocol. Cells were topped-up with 100 μl phenol-free Opti-MEM after 6 h.

Endogenous gene targeting (HEK293): stable reporter cells were seeded at 25,000 cells per well in 500 μl Opti-MEM on a 24-well plate. The next day, cells were transfected with a total of 1 μg pCas9/SYNE4 and/or pCas9/EMX1 plasmids using FuGENE (3 μl/μg). For RNP transfection, cells were transfected with RNP containing gRNA #3 targeting *SNCA* gene (Synthego) using Lipofectamine CRISPRMAX following manufacturer’s protocol.

Endogenous gene targeting (SH-SY5Y): stable repoter cells were seeded at 37,500 cells per well in 500 μl OptiMEM on a 24-well plate. The next day, cells were transfected with a total of 1 μg pCas9/gSNCA3 plasmid using Lipofectamine 2000 as per manufacturer’s protocol. For RNP transfection, cells were transfected with RNP containing gRNA #3 targeting *SNCA* gene using Lipofectamine RNAiMAX following manufacturer’s protocol.

Cells were topped up with 20% FBS containing Opti-MEM after 6 h. Cells were grown until they reached confluency and then transferred to a 6-well plate (Catalog# Nunc™145380, Thermo Fisher Scientific) and grown in complete DMEM until they reached confluency. Cells were further transferred to a T75 flask (Catalog # Nunc™156499, Thermo Fisher Scientific) before sorting.

### Microplate Reader Assay

To determine whether the microplate reader is sensitive enough to detect differences in cellular GFP expression, we seeded RFP^+^GFP^+^ expressing cells at defined cell numbers (1,000, 5,000, 10,000, 15,000, 20,000, and 30,000) in a 96-well plate (Catalog# 164588, Thermo Scientific Nunc Optical CVG, black-well, clear bottom) in a phenol-free medium to minimize fluorescence background (Supplementary Figure S2C). Cells were diluted with RFP^+^GFP^−^ cells to maintain a constant cell number of 40,000 per well and the plate was placed in the incubator overnight. 40,000 RFP^−^GFP^-^ were seeded and used as blank controls.

Fluorescence was measured using the filter-based multi-mode FLUOstar^®^ Omega microplate reader. Bottom optical scan was performed using Spiral Averaging or 10 × 10 Matrix Scan (6 mm diameter wells) to eliminate uneven cell distribution. Gain was determined using the microplate reader’s built-in gain adjustment option (RFP; 3,468, GFP; 2,259). Multiple scans were run to determine the optimal excitation and emission wavelengths (RFP; λ_EX_—584 nm, λ_EX_—620-10 nm, and GFP; λ_EX_—485-12 nm, λ_EX_—520 nm) based on signal to background ratio. Fluorescence intensities were corrected using signals obtained from RFP^−^GFP^-^ cells, and GFP/RFP ratios were calculated from normalized values.

By plotting normalized GFP/RFP ratios against % RFP^+^GFP^+^ cells counted using flowcytometry, the following formula was generated from the linear trendline:

$$\%RFP^{+}GFP^{+}=\frac{3\left(GFP/RFP\right)}{5}\times 100$$

To test and compare the assay with conventional flowcytometry, cells were transfected as described in Section 4.6. At the timepoints indicated in results Section 2.3, the plate was sealed using a transparent microplate sealer and read as described above. At 72 h, cells were additionally collected and analyzed using flowcytometry as described below.

### Flow Cytometry and Cell Sorting

HEK293 cells were dissociated by trypsinization for 5 min at 37°C and collected. Cells are filtered using a 50 μm cut-off cell strainer (BD Biosciences) to remove aggregates and collected in 5 ml round-bottom collection tubes. Flow cytometry was carried out in a LSRFortessa (BD Biosciences) machine, analyzing 10,000 events per sample for PE-CF594 (RFP) and Alexa-Fluor 488 (GFP) expression (Supplementary Figure S6A). The percentage of editing was then deduced by calculating the percentage of RFP^+^/GFP^+^ from all RFP^+^ cells.

Cell Sorting: After treatment with gRNA’s targeting the reporter gene and endogenous genes simultaneously, RFP^+^GFP^+^ and RFP^+^GFP^−^ cells were sorted using BD FACSAria II Cell Sorter (Supplementary Figure S6B). Cells were grown separately in 96-well plate and transferred to a 24-well plate and then to a 6-well plate. At confluency, cells were detached for DNA and protein analyses.

### DNA Extraction and PCR Amplification for the Genomic Target Genes

Genomic DNA was isolated from treated and untreated cells as control using QIAamp DNA Mini Kit (Catalog# 51306, Qiagen, Hilden, Germany) following the manufacturer’s protocol and protocol used by Aouida et al., 2015 (Aouida et al., 2015). PCR was carried out to amplify *SYNE4* target using AccuPrime High Fidelity Taq Polymerase (Catalog# 12346-086, Invitrogen, CA, United States), according to the manufacturer’s instructions for 40 cycles (94°C, 30 s; 65°C, 30 s; 68°C, 60 s). *EMX1* target was amplified using AccuPrime High Fidelity Taq Polymerase (annealing at 57°C). To amplify *SNCA* target, Phusion Hot Start Flex 2X Master Mix (NEB) was used, according to manufacturer’s instructions for 30 cycles (98°C, 30 s; 65°C, 35 s; 72°C, 30 s). Each mix was vortexed and centrifuged before placing into a thermal cycler. To confirm PCR, 2 μl of reaction mixed with 1ul loading dye (6X) and 3 μl H_2_O were loaded and run on a 2% agarose (Catalog# 16500100, Thermo Fisher Scientific) gel. Only one band of the expected size should show per sample. PCR products are then purified and eluted in water using Qiagen’s PCR purification kit manufacturer’s protocol.

### Western Blot

For measuring SNCA protein expression after enrichment of cells containing *SNCA* mutations, cells were suspended in lysis buffer (MT Cell Lysis Reagent, Catalog# CelLytic C3228-50ML, Sigma) containing 10 μl/ml Protease and Phosphatase Inhibitor Cocktail (Catalog# Halt™ 78440, Thermo Fisher Scientific) and lysed using manufacturer’s protocol. 30 μg cell lysates were loaded on 15% SDS polyacrylamide1 mm gel and separate by electrophoresis at 100 V for 90 min. Proteins were subsequently transferred onto a nitrocellulose membrane performed at 100 V for 60 min. The membrane was immediately placed in boiling PBS for 5 min, blocked with 5% blocking buffer (Blotting-grade blocker, Catalog#1706404, Biorad) in PBS-T for 1 h then incubated with 1:2,500 anti-α-synuclein antibody (Syn-1, Catalog# 610787, BD BioSciences) and β-actin antibody (ab8227, abcam) overnight at 4°C. The protein bands were visualized using Super Signal West Femto Chemiluminescent substrate (Thermo Scientific) and the band intensities determined using Quantity One-4.1.1 software (Bio-Rad) and ImageJ software (NIH, Bethesda, MD).

### Cytotoxicity Assay

To assess MPP + toxicity, we measured the cellular redox activity with MTT as previously described (Javed et al., 2016). Briefly, reporter SH-SY5Y cells were plated at a density of 5,000 cells per well on a 96-well plate in 100 µl complete media. The next day, cells were exposed to 1-methyl-4-phenyl pyridinium (MPP + iodide, Catalog# D048-100 MG, Sigma) for 24 h. MTT reagent (Catalog# M2128-1G, Sigma) were then added to a final concentration of 0.6 mg/ml and incubated for 4 h. Media was removed and 100 µl of cell lysis buffer, composed of15% SDS (Catalog# 11667289001, Sigma), 50% *N*,*N*-dimethylformamide (Catalog# 227056-100ML, Sigma), pH 4.7, was then added to the cells and incubated in a humidified incubator overnight at 37°C. Absorbance was then measured at 590 nm using a plate reader (TECAN).

## Data Availability

The original contributions presented in the study are included in the article/Supplementary Material, further inquiries can be directed to the corresponding authors.
