# Supplementary material for: Rapid Assessment of CRISPR Transfection Efficiency and Enrichment of CRISPR Induced Mutations Using a Dual-Fluorescent Stable Reporter System
Source: Front Genome Ed. 2022 Mar 21;4:854866. doi: 10.3389/fgeed.2022.854866 (PMC8978543; doi:10.3389/fgeed.2022.854866)
Supplement: Supplementary file 1 [file DataSheet1.docx]

**Supplementary Data**

**Rapid Assessment of CRISPR Transfection Efficiency and Enrichment of CRISPR Induced Mutations Using a Dual-Fluorescent Stable Reporter System**

**Karim E. Shalaby ^1,2^, Mustapha Aouida ^1,^*****, Vijay Gupta^2^, Simona S. Ghanem^2^, Omar M. A. El-Agnaf ^1,2,^***

^1^: Biological and Biomedical Sciences Division, College of Health & Life Sciences, Hamad Bin Khalifa University, Doha, Qatar

^2^: Neurological Disorders Research Center, Qatar Biomedical Research Institute (QBRI), Hamad Bin Khalifa University (HBKU), Qatar Foundation, Doha, Qatar

**Correspondence:*** MA: [maouida@hbku.edu.qa](mailto:maouida@hbku.edu.qa); OA: [oelagnaf@hbku.edu.qa](mailto:oelagnaf@hbku.edu.qa)

| Supplementary Fig. S1 | Analysis of genome editing using the stable reporter system in low expressing fluorescent reporter HEK293 cells |
| --- | --- |
| Supplementary Fig. S2 | Development of a microplate reader assay for quantifying CRISPR nuclease activity. |
| Supplementary Fig. S3 | Fluorescence assisted enrichment of SH-SY5Y cells treated with ribonucleoprotein targeting *SNCA* gene. |
| Supplementary Fig. S4 | Quantification of indels in sorted RFP^+^GFP^+^ cells using TIDE analysis. |
| Supplementary Fig. S5 | Comparison of nuclease activity with gRNAs targeting SNCA gene in SH-SY5Y cells. |
| Supplementary Fig. S6 | Flowcyomtery and fluorescence-assisted cell sorting gating strategy. |
| Supplementary Tables | Table 1; 2; 3: Primers, gRNAs and oligonucleotides used in this study |
| Additional Supplementary information | DNA Sanger’s sequences and maps of plasmids used in this study:  pMRS_Cas9 and pRG2S_Cas9 reporter, Cas9 expression plasmid constructs, DNA Sanger’s sequences |

**Figures:**

**Supplementary figure S1. Analysis of genome editing using the stable reporter system in low expressing fluorescent reporter HEK293 cells.** (A) A stable population of fluorescent reporter cells (~72% RFP) expressing the reporter construct were sorted using a cell sorter. (B) Reporter-gRNA2 was found to result in highest nuclease activity in high (left) and low (right) expressing stable reporter HEK293 cells using flow-cytometry 72 hours post-transfection of Cas9-expression plasmid and DNA fragments encoding each of the reporter-gRNAs expressed under a U6 promoter, using FuGENE. Results are expressed as mean ± standard error of the mean of *n* = 2. (C) Sample fluorescent microscopy images of low expressing stable reporter HEK293 cells cultured for 72 hours after transfection with a single plasmid co-expressing CRISPR-Cas9 protein and reporter-gRNA2 using FuGENE. Scale bar = 20µm.


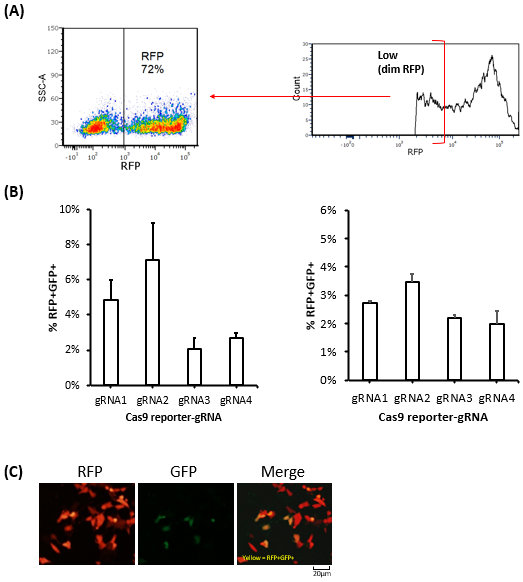


**Supplementary figure S2. Development of a microplate reader assay for quantifying CRISPR nuclease activity.** In duplicates, RFP^+^GFP^+^ cells were seeded at defined cell numbers and diluted with RFP^+^GFP^-^ cells to maintain a constant cell number of 40,000 per well in a 96-well plate and fluorescence intensity was measured after overnight incubation using FLUOstar^®^ Omega microplate reader. GFP Fluorescence intensity (A) and GFP/RFP ratio calculated using GFP and RFP fluorescence intensities (B) showed a linear relationship with increasing RFP^+^GFP^+^ cell numbers. (C) Cells were further analyzed under a fluorescence microscope and using flowcytometry. The percentage of RFP^+^GFP^+^ cells obtained using flowcytometry were plotted against GFP/RFP ratio and a standard equation (D) was generated from the linear trendline.

**
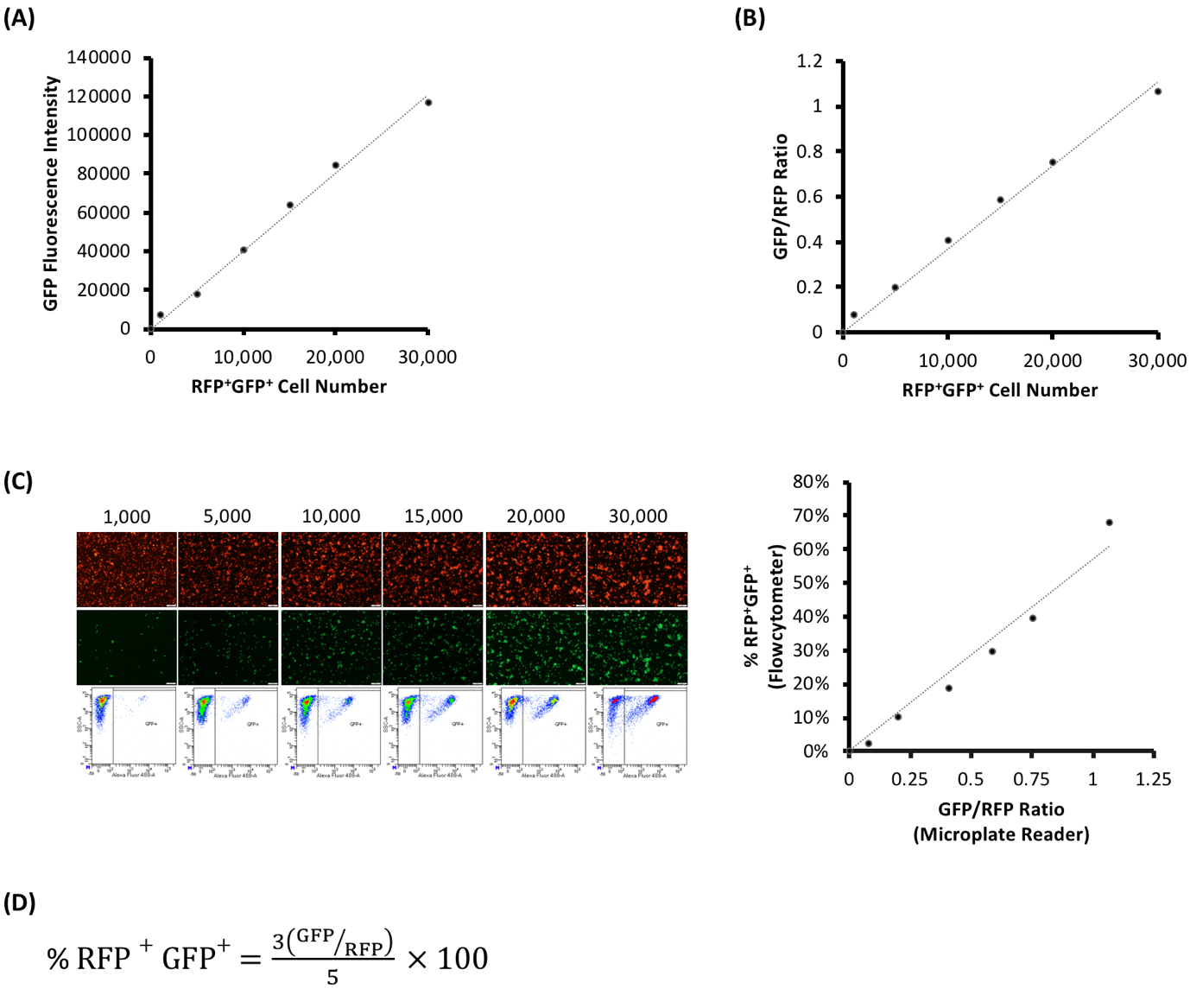
**

**Supplementary figure S3. Fluorescence assisted enrichment of SH-SY5Y cells treated with ribonucleoprotein targeting *SNCA* gene.** (A) Fluorescence Assisted Cell-Sorting (FACS) data showing the percentage of RFP^+^GFP^+^ cells obtained in *SYNE4*, *EMX1*, and *SNCA* targeted HEK293 stable reporter cells pre-sorting. (B) Microscopy images of pre- and post-sorted fluorescent reporter SH-SY5Y cells transfected with plasmid targeting *SNCA* gene. (C) T7 Endonuclease I (T7E1) assay carried out on genomic DNA extracted from post-sorted RFP+GFP- and RFP+GFP+ cells confirming the formation of indels in the target region. (D) Sanger’s sequencing of clones revealing CRISPR-induced indels and mutations. gRNA binding site (green) in the wildtype (WT) sequence is shown, the PAM sequence is underlined, dashes indicate deletions (-), nucleotides colored in blue indicate additions (+), and nucleotide colored in red indicate substitutions (s). Mutation frequencies are calculated as the number of clones carrying a mutation divided by the total number of clones sequenced. Quantification of indels from T7E1 is shown below.

**
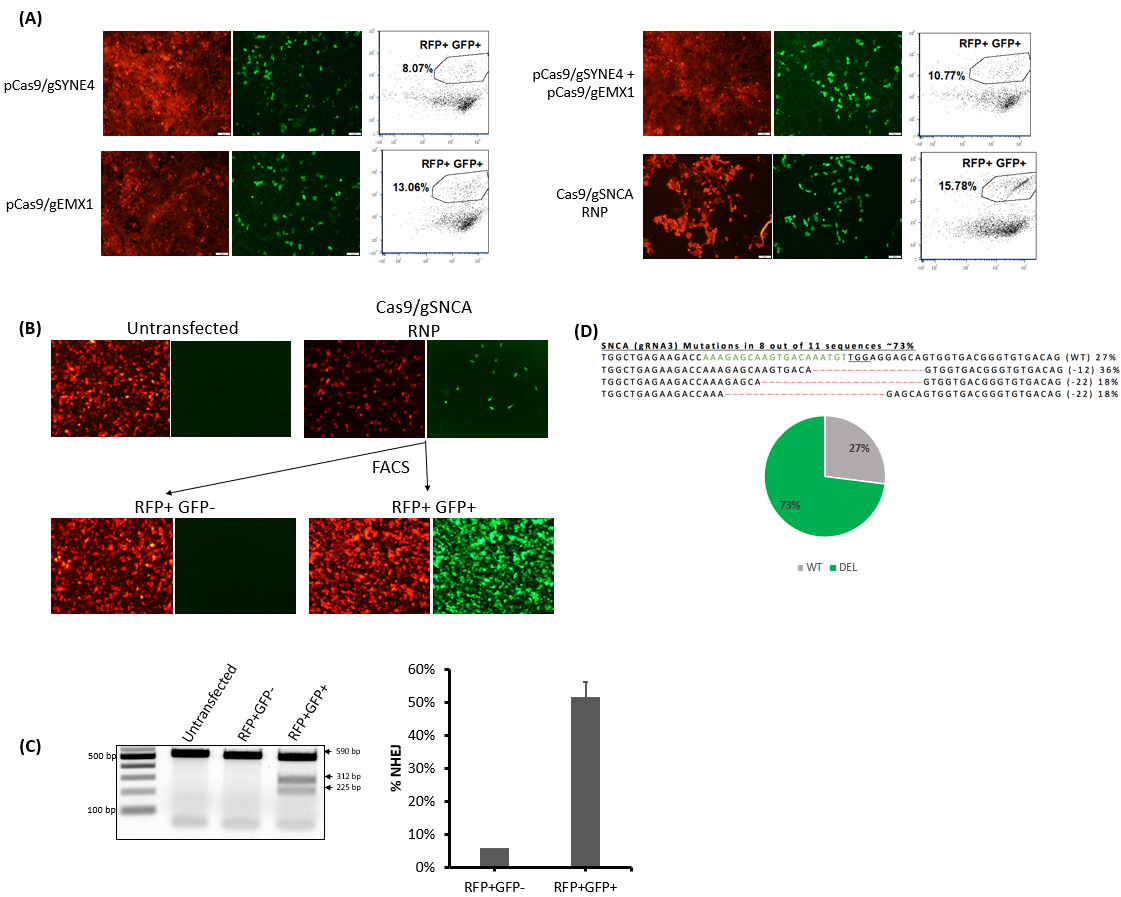
**

**Supplementary figure S4. Quantification of indels in sorted RFP^+^GFP^+^ cells using TIDE analysis.** TIDE analysis was performed using a reference sequence from untreated cells. (A) pCas9/gSYNE4 plasmid-treated HEK293 cells. (B) pCas9/gEMX1 plasmid-treated HEK293 cells. (C) Cas9/gSNCA4 RNP-treated HEK293 cells. (D) Cas9/gRNA4 RNP-treated SH-SY5Y cells. Left: Quantification of indel frequencies. Right: Quality control data showing indels formation at the gRNA target site.

**
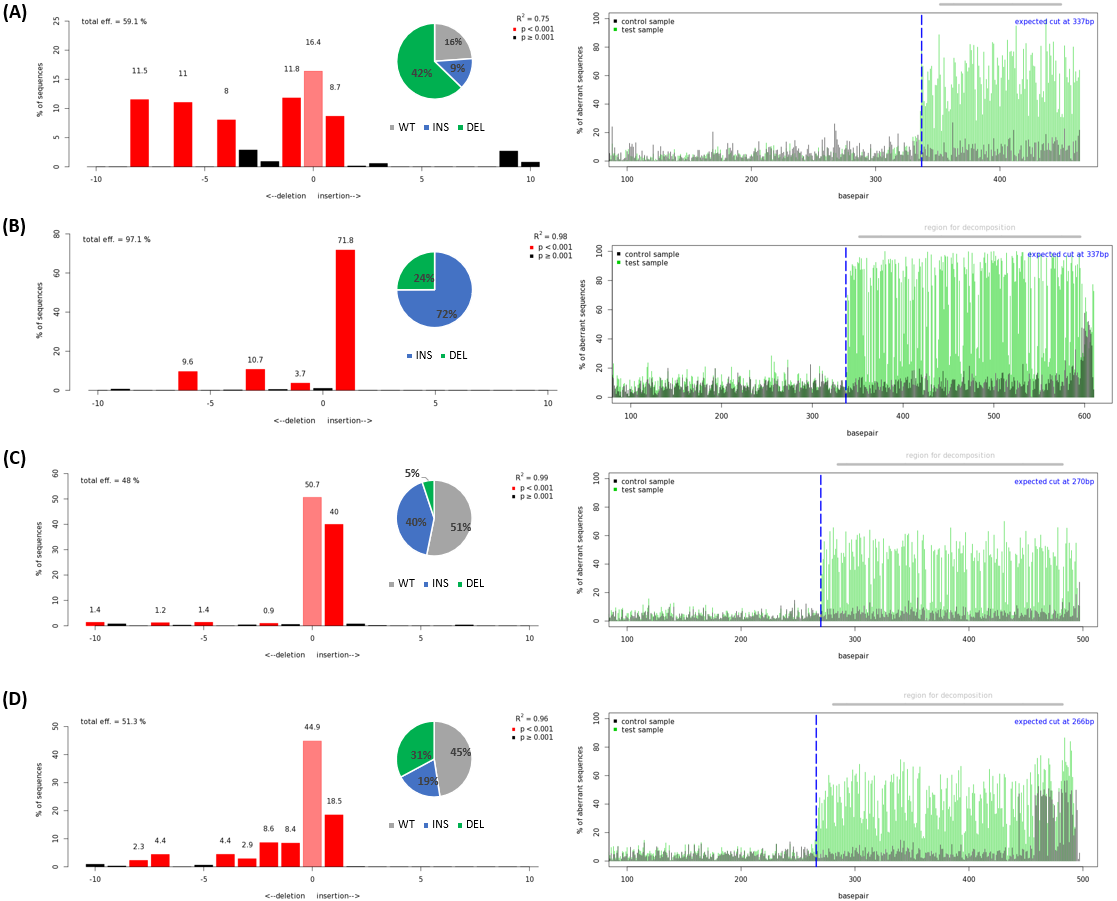
**

**Supplementary figure S5. Comparison of nuclease activity with gRNAs targeting *SNCA* gene in SH-SY5Y cells.** (A) T7 Endonuclease I (T7E1) assay carried out on genomic DNA extracted from SH-SY5Y cells transfected with ribonucleoprotein complex of Cas9 and either of four gRNAs targeting *SNCA* gene. The formation of indels in the target region was observed with highest activity attributed to gRNAs 1 and 3. Quantification of indels from T7E1 is shown on the right. (B) Western blot evaluation of endogenous α-synuclein protein expression. Quantification of protein expression from western blot is shown on the right. (UT = untransfected).


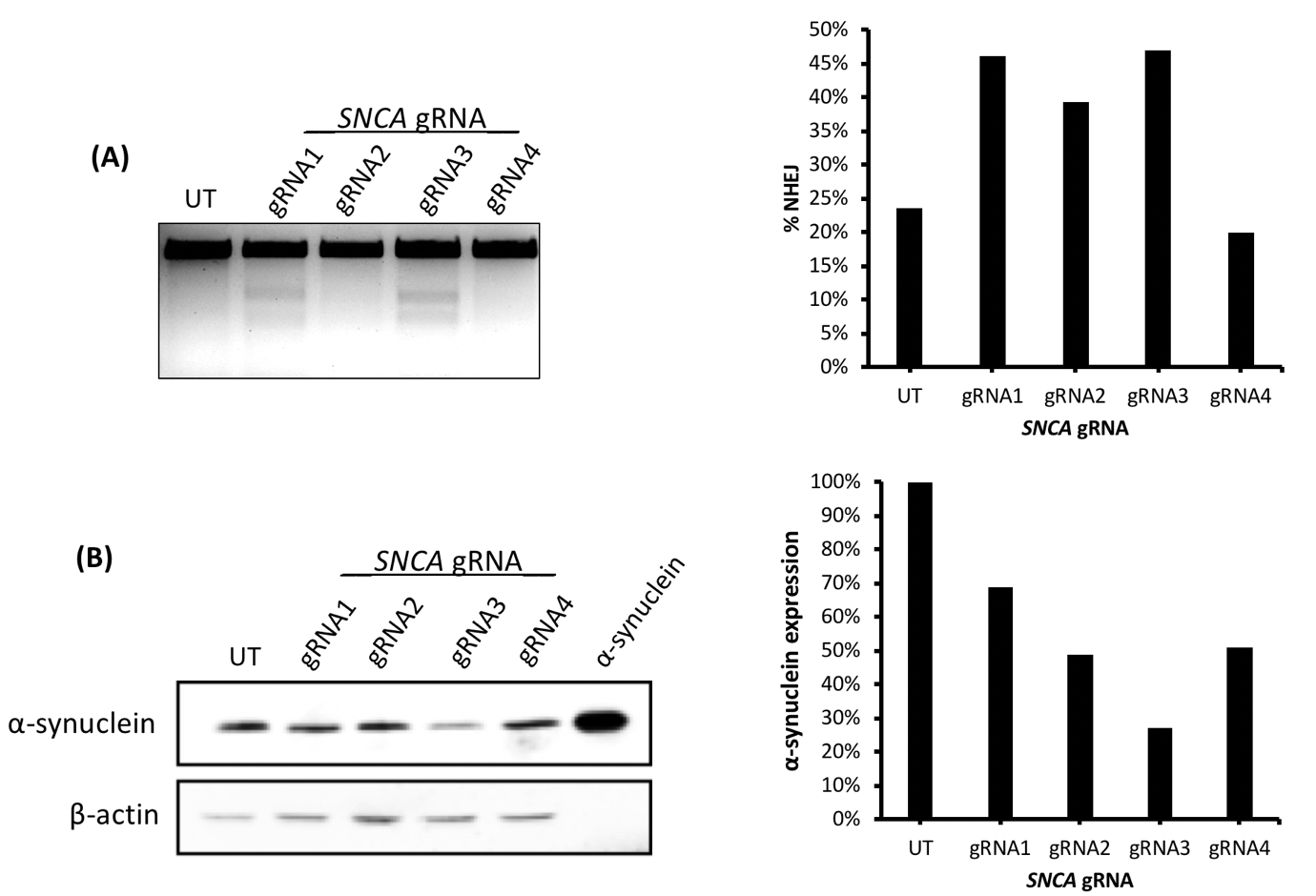


**Supplementary figure S6. Flowcytomtery and fluorescence-assisted cell sorting gating strategy.** (A) Gating of representative Cas9/reporter-gRNA treated cells in the order of Cells > Singlets > Viable >RFP^+^/GFP^+^ using BD LSRFortessa flowcytometer. (B) Gating of representative cells treated with Cas9/reporter-gRNA+gRNA targeting endogenous genes in the order of Cells > Singlets > RFP+ > GFP^+^ using BD FACSAria II Cell Sorter for enrichment of cells containing target mutations. Data were analyzed using FCS Express Cytometry 7 software.


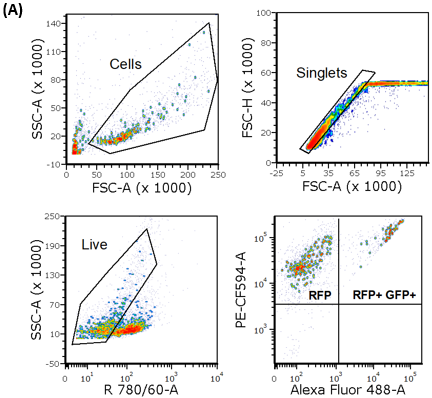

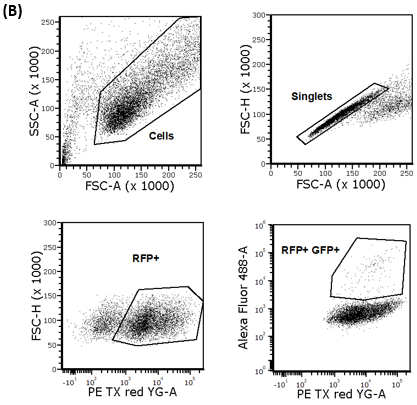


**Tables:**

**Table 1: Primers used for cloning, sequencing and amplification of gRNAs:**

| Primer | Sequence | Experiment |
| --- | --- | --- |
| pRG2S_Forward | 5’-CTGCCCGGCGCCTACAAGAC-3’ | Cloning Cas9 target sequence |
| pRG2S_Reverse | 5’-CGTAGCCTTCGGGCATGGCG-3’ |  |
| SYNE4_Forward | 5’-CTGCAGCCTCAGGCCCGGGT-3’ | Amplifying SYNE4 target sequence for mutation analysis |
| SYNE4_Reverse | 5’- GGCCCACCCTGGAAGTGCTCAGG-3’ |  |
| EMX1_Forward | 5’- GAACAGGAAAACCACCCTTCTCTC -3’ | Amplifying EMX1 target sequence for mutation analysis |
| EMX1_Reverse | 5’- CAGCCAGCCCATTGCTTGTC -3’ |  |
| SNCA_Forward | 5’-CACTCATGGCTTTACATTCCTGATCGT-3’ | Amplifying SNCA target sequence for mutation analysis |
| SNCA_Reverse | 5’-TCAGGTAGCCGTTCCCCACAGTAA-3’ |  |
| M13_Forward (-20) | 5’-GTAAAACGACGGCCAG-3’ | Sanger's sequencing of the edited gene targets |
| U6-gRNA_Forward | 5’-GCGGCCGCGAGGGCCTATTT-3’ | Amplifying U6 gRNA expression fragments |
| U6-gRNA_Reverse | 5’- GGATCCTAGTACTCGAGAAAAAAAGCACCG-3’ |  |

**Table 2: Guide RNAs (gRNAs) used for targeting reporter and endogenous genes:**

| gRNA | Sequence | Experiment |
| --- | --- | --- |
| Cas9_Target_gRNA1 | 5’-CATCTACAAGCAGTCACAGC-3’ | Reporter assay for the detection of CRISPR-Cas9 activity |
| Cas9_Target_gRNA2 | 5’-CTGCTGTGACTGCTTGTAGA-3’ | Reporter assay for the detection of CRISPR-Cas9 activity |
| Cas9_Target_gRNA3 | 5’-TGACTGCTTGTAGATGGCCA-3’ | Reporter assay for the detection of CRISPR-Cas9 activity |
| Cas9_Target_gRNA4 | 5’-CCGAATTCGCGTCCGCGCCA-3’ | Reporter assay for the detection of CRISPR-Cas9 activity |
| U6-pRG2S-Cas9-gRNA1 | GCGGCCGCGAGGGCCTATTTCCCATGATTCCTTCATATTTGCATATACGATACAAGGCTGTTAGAGAGATAATTAGAATTAATTTGACTGTAAACACAAAGATATTAGTACAAAATACGTGACGTAGAAAGTAATAATTTCTTGGGTAGTTTGCAGTTTTAAAATTATGTTTTAAAATGGACTATCATATGCTTACCGTAACTTGAAAGTATTTCGATTTCTTGGGTTTATATATCTTGTGGAAAGGACCCACCTTGTTGGCATCTACAAGCAGTCACAGCGTTTTAGAGCTAGAAATAGCAAGTTAAAATAAGGCTAGTCCGTTATCAACTTGAAAAAGTGGCACCGAGTCGGTGCTTTTTTTCTCGAGTACTAGGATCC | Reporter assay for the detection of CRISPR-Cas9 activity |
| U6-pRG2S-Cas9-gRNA2 | GCGGCCGCGAGGGCCTATTTCCCATGATTCCTTCATATTTGCATATACGATACAAGGCTGTTAGAGAGATAATTAGAATTAATTTGACTGTAAACACAAAGATATTAGTACAAAATACGTGACGTAGAAAGTAATAATTTCTTGGGTAGTTTGCAGTTTTAAAATTATGTTTTAAAATGGACTATCATATGCTTACCGTAACTTGAAAGTATTTCGATTTCTTGGGTTTATATATCTTGTGGAAAGGACCCACCTTGTTGGCTGCTGTGACTGCTTGTAGAGTTTTAGAGCTAGAAATAGCAAGTTAAAATAAGGCTAGTCCGTTATCAACTTGAAAAAGTGGCACCGAGTCGGTGCTTTTTTTCTCGAGTACTAGGATCC | Reporter assay for the detection of CRISPR-Cas9 activity |
| U6-pRG2S-Cas9-gRNA3 | GCGGCCGCGAGGGCCTATTTCCCATGATTCCTTCATATTTGCATATACGATACAAGGCTGTTAGAGAGATAATTAGAATTAATTTGACTGTAAACACAAAGATATTAGTACAAAATACGTGACGTAGAAAGTAATAATTTCTTGGGTAGTTTGCAGTTTTAAAATTATGTTTTAAAATGGACTATCATATGCTTACCGTAACTTGAAAGTATTTCGATTTCTTGGGTTTATATATCTTGTGGAAAGGACCCACCTTGTTGGTGACTGCTTGTAGATGGCCAGTTTTAGAGCTAGAAATAGCAAGTTAAAATAAGGCTAGTCCGTTATCAACTTGAAAAAGTGGCACCGAGTCGGTGCTTTTTTTCTCGAGTACTAGGATCC | Reporter assay for the detection of CRISPR-Cas9 activity |
| U6-pRG2S-Cas9-gRNA4 | GCGGCCGCGAGGGCCTATTTCCCATGATTCCTTCATATTTGCATATACGATACAAGGCTGTTAGAGAGATAATTAGAATTAATTTGACTGTAAACACAAAGATATTAGTACAAAATACGTGACGTAGAAAGTAATAATTTCTTGGGTAGTTTGCAGTTTTAAAATTATGTTTTAAAATGGACTATCATATGCTTACCGTAACTTGAAAGTATTTCGATTTCTTGGGTTTATATATCTTGTGGAAAGGACCCACCTTGTTGGCCGAATTCGCGTCCGCGCCAGTTTTAGAGCTAGAAATAGCAAGTTAAAATAAGGCTAGTCCGTTATCAACTTGAAAAAGTGGCACCGAGTCGGTGCTTTTTTTCTCGAGTACTAGGATCC | Reporter assay for the detection of CRISPR-Cas9 activity |
| gSYNE4 crRNA | 5’-CATGGCCCTGTCCCTGCCTC-3’ | Inducing mutation in *SYNE4* gene |
| gEMX1 crRNA | 5’-GAGTCCGAGCAGAAGAAGAA-3’ | Inducing mutation in *EMX1* gene |
| gSNCA1 sgRNA | 5’-GACAAATGTTGGAGGAGCAG-3’ | Inducing mutation in and knocking out *SNCA* gene |
| gSNCA2 sgRNA | 5’- GAGCAAGTGACAAATGTTGG-3’ | Inducing mutation in and knocking out *SNCA* gene |
| gSNCA3 sgRNA | 5’- AAAGAGCAAGTGACAAATGT-3’ | Inducing mutation in and knocking out *SNCA* gene |
| gSNCA4 crRNA | 5’- AACATTTGTCACTTGCTCTT-3’ | Inducing mutation in and knocking out *SNCA* gene |

**Table 3: Oligonucleotides used for cloning *SNCA* gRNAs into Cas9 expression plasmid:**

| Oligonucleotide | Sequence | Experiment |
| --- | --- | --- |
| gSNCA1_sense | 5'-caccGGTGGATGACATTCTGGAGC-'3 | Constructing pCas9/gSNCA1 plasmid |
| gSNCA1_antisense | 5'-aaacGCTCCAGAATGTCATCCACC-'3 |  |
| gSNCA3_sense | 5’-caccAAAGAGCAAGTGACAAATGT-3’ | Constructing pCas9/gSNCA3 plasmid |
| gSNCA3_antisense | 5’-aaacGACAAATGTTGGAGGAGCAG-3’ |  |

**DNA sequences and maps of plasmids used in this study:**

**Cas9 Target Sequence: (yellow, cloned between BamHI and BstXI)**

5’-atggccaagaagcccgtgcagctgcccggcgcctacaagaccgacatcaagctggacatcacctcccacaacgaggactacaccatcgtggaacagtacgagcgcgccgagggccgccactccaccggcgccgaattcGCGTCCGCGCCATGGCCATCTACAAGCAGTCACAGCAg-3’

**pMRS_Cas9 sequence and map:**

tagttattaatagtaatcaattacggggtcattagttcatagcccatatatggagttccgcgttacataacttacggtaaatggcccgcctggctgaccgcccaacgacccccgcccattgacgtcaataatgacgtatgttcccatagtaacgccaatagggactttccattgacgtcaatgggtggagtatttacggtaaactgcccacttggcagtacatcaagtgtatcatatgccaagtacgccccctattgacgtcaatgacggtaaatggcccgcctggcattatgcccagtacatgaccttatgggactttcctacttggcagtacatctacgtattagtcatcgctattaccatggtgatgcggttttggcagtacatcaatgggcgtggatagcggtttgactcacggggatttccaagtctccaccccattgacgtcaatgggagtttgttttggcaccaaaatcaacgggactttccaaaatgtcgtaacaactccgccccattgacgcaaatgggcggtaggcgtgtacggtgggaggtctatataagcagagctggtttagtgaaccgtcagatccgcttgccaccatggcctcctccgaggacgtcatcaaggagttcatgcgcttcaaggtgcgcatggagggctccgtgaacggccacgagttcgagatcgagggcgagggcgagggccgcccctacgagggcacccagaccgccaagctgaaggtgaccaagggcggccccctgcccttcgcctgggacatcctgtcccctcagttccagtacggctccaaggcctacgtgaagcaccccgccgacatccccgactacttgaagctgtccttccccgagggcttcaagtgggagcgcgtgatgaacttcgaggacggcggcgtggtgaccgtgacccaggactcctccctgcaggacggcgagttcatctacaaggtgaagctgcgcggcaccaacttcccctccgacggccccgtaatgcagaagaagaccatgggctgggaggcctccaccgagcggatgtaccccgaggacggcgccctgaagggcgagatcaagatgaggctgaagctgaaggacggcggccactacgacgccgaggtcaagaccacctacatggccaagaagcccgtgcagctgcccggcgcctacaagaccgacatcaagctggacatcacctcccacaacgaggactacaccatcgtggaacagtacgagcgcgccgagggccgccactccaccggcgccgaattcGCGTCCGCGCCATGGCCATCTACAAGCAGTCACAGCAggatccagtgagcaagggcgaggagctgttcaccggggtggtgcccatcctggtcgagctggacggcgacgtaaacggccacaagttcagcgtgtccggcgagggcgagggcgatgccacctacggcaagctgaccctgaagttcatctgcaccaccggcaagctgcccgtgccctggcccaccctcgtgaccaccctgacctacggcgtgcagtgcttcagccgctaccccgaccacatgaagcagcacgacttcttcaagtccgccatgcccgaaggctacgtccaggagcgcaccatcttcttcaaggacgacggcaactacaagacccgcgccgaggtgaagttcgagggcgacaccctggtgaaccgcatcgagctgaagggcatcgacttcaaggaggacggcaacatcctggggcacaagctggagtacaactacaacagccacaacgtctatatcatggccgacaagcagaagaacggcatcaaggtgaacttcaagatccgccacaacatcgaggacggcagcgtgcagctcgccgaccactaccagcagaacacccccatcggcgacggccccgtgctgctgcccgacaaccactacctgagcacccagtccgccctgagcaaagaccccaacgagaagcgcgatcacatggtcctgctggagttcgtgaccgccgccgggatcactctcggcatggacgagctgtacaagcaatgtactaactacgctttgttgaaactcgctggcgatgttgaaagtaaccccggtcctgctagcatggcaccctgcatgctgctcctgctgttggcggccgccctggccccgactcagacccgcgcgggcccacattcgctgaggtatttccacaccgccgtgtcccggcccggcctcgggaagccccggttcatctctgtcggctacgtggacgacacgcagttcgtgcgcttcgacagcgacgcggagaatccgaggtatgagccgcgggtgcggtggatggagcaggtggagcccgagtattgggagcggaacacgcagatcgccaagggcaatgagcagattttccgagtgaacctgaggaccgcgctgcgctactacaaccagagcgcgggcggctctcacacgttccaacggatgtacggctgtgaggtggggtcggactggcgcctcctccgcgggtacgagcagtacgcatacgacggctgcgattacatcgccctgaacgaagacctgaaaacgtggacggcggccgacatggcggcgctgatcaccaaacacaagtgggagcaggctggtgatgcagagagagaccgggcctacctggagggcacgtgcgtggagtggctccgcagatacctgcagctcgggaacgcgacgctgccgcgcacagattccccaaaggcccatgtgacccgtcacagcagacctgaagataaagtcaccctgaggtgctgggccctgggcttctaccctgctgacatcaccctgacctggcagttgaatggggaggagctgacccaggacatggagcttgtggagaccaggcctgcaggggatggaaccttccagaagtgggcatctgtggtggtgcctcttgggaaggagcagtattacacatgccatgtgtaccatcaggggctgcctgagcccctcaccctgagatgggagcctcctccatccactgtctccaacacggtaatcattgctgttctggttgtccttggagctgcaatagtcactggagctgtggtggcttttgtgatgaagatgagaaggagaaacacaggtggaaaaggagggtaagcggccgcgactctagatcataatcagccataccacatttgtagaggttttacttgctttaaaaaacctcccacacctccccctgaacctgaaacataaaatgaatgcaattgttgttgttaacttgtttattgcagcttataatggttacaaataaagcaatagcatcacaaatttcacaaataaagcatttttttcactgcattctagttgtggtttgtccaaactcatcaatgtatcttaaggcgtaaattgtaagcgttaatattttgttaaaattcgcgttaaatttttgttaaatcagctcattttttaaccaataggccgaaatcggcaaaatcccttataaatcaaaagaatagaccgagatagggttgagtgttgttccagtttggaacaagagtccactattaaagaacgtggactccaacgtcaaagggcgaaaaaccgtctatcagggcgatggcccactacgtgaaccatcaccctaatcaagttttttggggtcgaggtgccgtaaagcactaaatcggaaccctaaagggagcccccgatttagagcttgacggggaaagccggcgaacgtggcgagaaaggaagggaagaaagcgaaaggagcgggcgctagggcgctggcaagtgtagcggtcacgctgcgcgtaaccaccacacccgccgcgcttaatgcgccgctacagggcgcgtcaggtggcacttttcggggaaatgtgcgcggaacccctatttgtttatttttctaaatacattcaaatatgtatccgctcatgagacaataaccctgataaatgcttcaataatattgaaaaaggaagagtcctgaggcggaaagaaccagctgtggaatgtgtgtcagttagggtgtggaaagtccccaggctccccagcaggcagaagtatgcaaagcatgcatctcaattagtcagcaaccaggtgtggaaagtccccaggctccccagcaggcagaagtatgcaaagcatgcatctcaattagtcagcaaccatagtcccgcccctaactccgcccatcccgcccctaactccgcccagttccgcccattctccgccccatggctgactaattttttttatttatgcagaggccgaggccgcctcggcctctgagctattccagaagtagtgaggaggcttttttggaggcctaggcttttgcaaagatcgatcaagagacaggatgaggatcgtttcgcatgattgaacaagatggattgcacgcaggttctccggccgcttgggtggagaggctattcggctatgactgggcacaacagacaatcggctgctctgatgccgccgtgttccggctgtcagcgcaggggcgcccggttctttttgtcaagaccgacctgtccggtgccctgaatgaactgcaagacgaggcagcgcggctatcgtggctggccacgacgggcgttccttgcgcagctgtgctcgacgttgtcactgaagcgggaagggactggctgctattgggcgaagtgccggggcaggatctcctgtcatctcaccttgctcctgccgagaaagtatccatcatggctgatgcaatgcggcggctgcatacgcttgatccggctacctgcccattcgaccaccaagcgaaacatcgcatcgagcgagcacgtactcggatggaagccggtcttgtcgatcaggatgatctggacgaagagcatcaggggctcgcgccagccgaactgttcgccaggctcaaggcgagcatgcccgacggcgaggatctcgtcgtgacccatggcgatgcctgcttgccgaatatcatggtggaaaatggccgcttttctggattcatcgactgtggccggctgggtgtggcggaccgctatcaggacatagcgttggctacccgtgatattgctgaagagcttggcggcgaatgggctgaccgcttcctcgtgctttacggtatcgccgctcccgattcgcagcgcatcgccttctatcgccttcttgacgagttcttctgagcgggactctggggttcgaaatgaccgaccaagcgacgcccaacctgccatcacgagatttcgattccaccgccgccttctatgaaaggttgggcttcggaatcgttttccgggacgccggctggatgatcctccagcgcggggatctcatgctggagttcttcgcccaccctagggggaggctaactgaaacacggaaggagacaataccggaaggaacccgcgctatgacggcaataaaaagacagaataaaacgcacggtgttgggtcgtttgttcataaacgcggggttcggtcccagggctggcactctgtcgataccccaccgagaccccattggggccaatacgcccgcgtttcttccttttccccaccccaccccccaagttcgggtgaaggcccagggctcgcagccaacgtcggggcggcaggccctgccatagcctcaggttactcatatatactttagattgatttaaaacttcatttttaatttaaaaggatctaggtgaagatcctttttgataatctcatgaccaaaatcccttaacgtgagttttcgttccactgagcgtcagaccccgtagaaaagatcaaaggatcttcttgagatcctttttttctgcgcgtaatctgctgcttgcaaacaaaaaaaccaccgctaccagcggtggtttgtttgccggatcaagagctaccaactctttttccgaaggtaactggcttcagcagagcgcagataccaaatactgtccttctagtgtagccgtagttaggccaccacttcaagaactctgtagcaccgcctacatacctcgctctgctaatcctgttaccagtggctgctgccagtggcgataagtcgtgtcttaccgggttggactcaagacgatagttaccggataaggcgcagcggtcgggctgaacggggggttcgtgcacacagcccagcttggagcgaacgacctacaccgaactgagatacctacagcgtgagctatgagaaagcgccacgcttcccgaagggagaaaggcggacaggtatccggtaagcggcagggtcggaacaggagagcgcacgagggagcttccagggggaaacgcctggtatctttatagtcctgtcgggtttcgccacctctgacttgagcgtcgatttttgtgatgctcgtcaggggggcggagcctatggaaaaacgccagcaacgcggcctttttacggttcctggccttttgctggccttttgctcacatgttctttcctgcgttatcccctgattctgtggataaccgtattaccgccatgcat


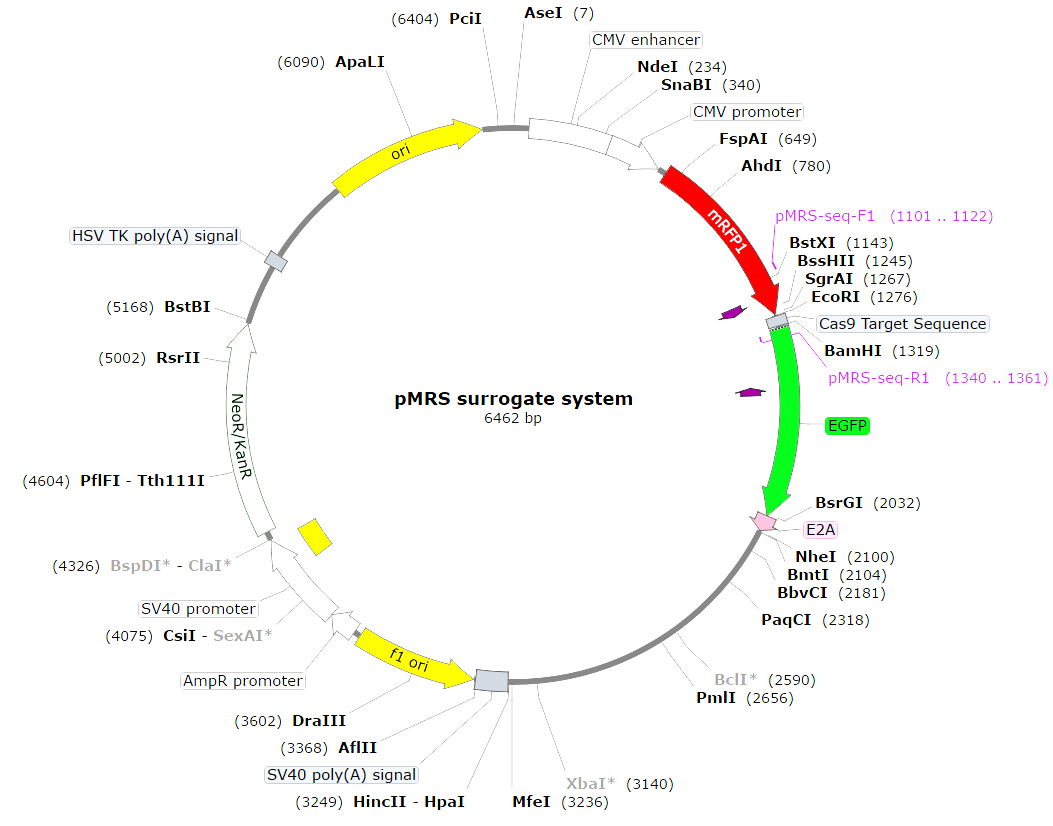


**pRG2S_Cas9 sequence and map:**

tagttattaatagtaatcaattacggggtcattagttcatagcccatatatggagttccgcgttacataacttacggtaaatggcccgcctggctgaccgcccaacgacccccgcccattgacgtcaataatgacgtatgttcccatagtaacgccaatagggactttccattgacgtcaatgggtggagtatttacggtaaactgcccacttggcagtacatcaagtgtatcatatgccaagtacgccccctattgacgtcaatgacggtaaatggcccgcctggcattatgcccagtacatgaccttatgggactttcctacttggcagtacatctacgtattagtcatcgctattaccatggtgatgcggttttggcagtacatcaatgggcgtggatagcggtttgactcacggggatttccaagtctccaccccattgacgtcaatgggagtttgttttggcaccaaaatcaacgggactttccaaaatgtcgtaacaactccgccccattgacgcaaatgggcggtaggcgtgtacggtgggaggtctatataagcagagctggtttagtgaaccgtcagatccgctagccaccatggcctcctccgaggacgtcatcaaggagttcatgcgcttcaaggtgcgcatggagggctccgtgaacggccacgagttcgagatcgagggcgagggcgagggccgcccctacgagggcacccagaccgccaagctgaaggtgaccaagggcggccccctgcccttcgcctgggacatcctgtcccctcagttccagtacggctccaaggcctacgtgaagcaccccgccgacatccccgactacttgaagctgtccttccccgagggcttcaagtgggagcgcgtgatgaacttcgaggacggcggcgtggtgaccgtgacccaggactcctccctgcaggacggcgagttcatctacaaggtgaagctgcgcggcaccaacttcccctccgacggccccgtaatgcagaagaagaccatgggctgggaggcctccaccgagcggatgtaccccgaggacggcgccctgaagggcgagatcaagatgaggctgaagctgaaggacggcggccactacgacgccgaggtcaagaccacctacatggccaagaagcccgtgcagctgcccggcgcctacaagaccgacatcaagctggacatcacctcccacaacgaggactacaccatcgtggaacagtacgagcgcgccgagggccgccactccaccggcgccgaattcGCGTCCGCGCCATGGCCATCTACAAGCAGTCACAGCAggatccagtgagcaagggcgaggagctgttcaccggggtggtgcccatcctggtcgagctggacggcgacgtaaacggccacaagttcagcgtgtccggcgagggcgagggcgatgccacctacggcaagctgaccctgaagttcatctgcaccaccggcaagctgcccgtgccctggcccaccctcgtgaccaccctgacctacggcgtgcagtgcttcagccgctaccccgaccacatgaagcagcacgacttcttcaagtccgccatgcccgaaggctacgtccaggagcgcaccatcttcttcaaggacgacggcaactacaagacccgcgccgaggtgaagttcgagggcgacaccctggtgaaccgcatcgagctgaagggcatcgacttcaaggaggacggcaacatcctggggcacaagctggagtacaactacaacagccacaacgtctatatcatggccgacaagcagaagaacggcatcaaggtgaacttcaagatccgccacaacatcgaggacggcagcgtgcagctcgccgaccactaccagcagaacacccccatcggcgacggccccgtgctgctgcccgacaaccactacctgagcacccagtccgccctgagcaaagaccccaacgagaagcgcgatcacatggtcctgctggagttcgtgaccgccgccgggatcactctcggcatggacgagctgtacaagtaaagcggccgccagtgagcaagggcgaggagctgttcaccggggtggtgcccatcctggtcgagctggacggcgacgtaaacggccacaagttcagcgtgtccggcgagggcgagggcgatgccacctacggcaagctgaccctgaagttcatctgcaccaccggcaagctgcccgtgccctggcccaccctcgtgaccaccctgacctacggcgtgcagtgcttcagccgctaccccgaccacatgaagcagcacgacttcttcaagtccgccatgcccgaaggctacgtccaggagcgcaccatcttcttcaaggacgacggcaactacaagacccgcgccgaggtgaagttcgagggcgacaccctggtgaaccgcatcgagctgaagggcatcgacttcaaggaggacggcaacatcctggggcacaagctggagtacaactacaacagccacaacgtctatatcatggccgacaagcagaagaacggcatcaaggtgaacttcaagatccgccacaacatcgaggacggcagcgtgcagctcgccgaccactaccagcagaacacccccatcggcgacggccccgtgctgctgcccgacaaccactacctgagcacccagtccgccctgagcaaagaccccaacgagaagcgcgatcacatggtcctgctggagttcgtgaccgccgccgggatcactctcggcatggacgagctgtacaagtaagcggccgcgactctagatcataatcagccataccacatttgtagaggttttacttgctttaaaaaacctcccacacctccccctgaacctgaaacataaaatgaatgcaattgttgttgttaacttgtttattgcagcttataatggttacaaataaagcaatagcatcacaaatttcacaaataaagcatttttttcactgcattctagttgtggtttgtccaaactcatcaatgtatcttaaggcgtaaattgtaagcgttaatattttgttaaaattcgcgttaaatttttgttaaatcagctcattttttaaccaataggccgaaatcggcaaaatcccttataaatcaaaagaatagaccgagatagggttgagtgttgttccagtttggaacaagagtccactattaaagaacgtggactccaacgtcaaagggcgaaaaaccgtctatcagggcgatggcccactacgtgaaccatcaccctaatcaagttttttggggtcgaggtgccgtaaagcactaaatcggaaccctaaagggagcccccgatttagagcttgacggggaaagccggcgaacgtggcgagaaaggaagggaagaaagcgaaaggagcgggcgctagggcgctggcaagtgtagcggtcacgctgcgcgtaaccaccacacccgccgcgcttaatgcgccgctacagggcgcgtcaggtggcacttttcggggaaatgtgcgcggaacccctatttgtttatttttctaaatacattcaaatatgtatccgctcatgagacaataaccctgataaatgcttcaataatattgaaaaaggaagagtcctgaggcggaaagaaccagctgtggaatgtgtgtcagttagggtgtggaaagtccccaggctccccagcaggcagaagtatgcaaagcatgcatctcaattagtcagcaaccaggtgtggaaagtccccaggctccccagcaggcagaagtatgcaaagcatgcatctcaattagtcagcaaccatagtcccgcccctaactccgcccatcccgcccctaactccgcccagttccgcccattctccgccccatggctgactaattttttttatttatgcagaggccgaggccgcctcggcctctgagctattccagaagtagtgaggaggcttttttggaggcctaggcttttgcaaagatcgatcaagagacaggatgaggatcgtttcgcatgattgaacaagatggattgcacgcaggttctccggccgcttgggtggagaggctattcggctatgactgggcacaacagacaatcggctgctctgatgccgccgtgttccggctgtcagcgcaggggcgcccggttctttttgtcaagaccgacctgtccggtgccctgaatgaactgcaagacgaggcagcgcggctatcgtggctggccacgacgggcgttccttgcgcagctgtgctcgacgttgtcactgaagcgggaagggactggctgctattgggcgaagtgccggggcaggatctcctgtcatctcaccttgctcctgccgagaaagtatccatcatggctgatgcaatgcggcggctgcatacgcttgatccggctacctgcccattcgaccaccaagcgaaacatcgcatcgagcgagcacgtactcggatggaagccggtcttgtcgatcaggatgatctggacgaagagcatcaggggctcgcgccagccgaactgttcgccaggctcaaggcgagcatgcccgacggcgaggatctcgtcgtgacccatggcgatgcctgcttgccgaatatcatggtggaaaatggccgcttttctggattcatcgactgtggccggctgggtgtggcggaccgctatcaggacatagcgttggctacccgtgatattgctgaagagcttggcggcgaatgggctgaccgcttcctcgtgctttacggtatcgccgctcccgattcgcagcgcatcgccttctatcgccttcttgacgagttcttctgagcgggactctggggttcgaaatgaccgaccaagcgacgcccaacctgccatcacgagatttcgattccaccgccgccttctatgaaaggttgggcttcggaatcgttttccgggacgccggctggatgatcctccagcgcggggatctcatgctggagttcttcgcccaccctagggggaggctaactgaaacacggaaggagacaataccggaaggaacccgcgctatgacggcaataaaaagacagaataaaacgcacggtgttgggtcgtttgttcataaacgcggggttcggtcccagggctggcactctgtcgataccccaccgagaccccattggggccaatacgcccgcgtttcttccttttccccaccccaccccccaagttcgggtgaaggcccagggctcgcagccaacgtcggggcggcaggccctgccatagcctcaggttactcatatatactttagattgatttaaaacttcatttttaatttaaaaggatctaggtgaagatcctttttgataatctcatgaccaaaatcccttaacgtgagttttcgttccactgagcgtcagaccccgtagaaaagatcaaaggatcttcttgagatcctttttttctgcgcgtaatctgctgcttgcaaacaaaaaaaccaccgctaccagcggtggtttgtttgccggatcaagagctaccaactctttttccgaaggtaactggcttcagcagagcgcagataccaaatactgtccttctagtgtagccgtagttaggccaccacttcaagaactctgtagcaccgcctacatacctcgctctgctaatcctgttaccagtggctgctgccagtggcgataagtcgtgtcttaccgggttggactcaagacgatagttaccggataaggcgcagcggtcgggctgaacggggggttcgtgcacacagcccagcttggagcgaacgacctacaccgaactgagatacctacagcgtgagctatgagaaagcgccacgcttcccgaagggagaaaggcggacaggtatccggtaagcggcagggtcggaacaggagagcgcacgagggagcttccagggggaaacgcctggtatctttatagtcctgtcgggtttcgccacctctgacttgagcgtcgatttttgtgatgctcgtcaggggggcggagcctatggaaaaacgccagcaacgcggcctttttacggttcctggccttttgctggccttttgctcacatgttctttcctgcgttatcccctgattctgtggataaccgtattaccgccatgcat


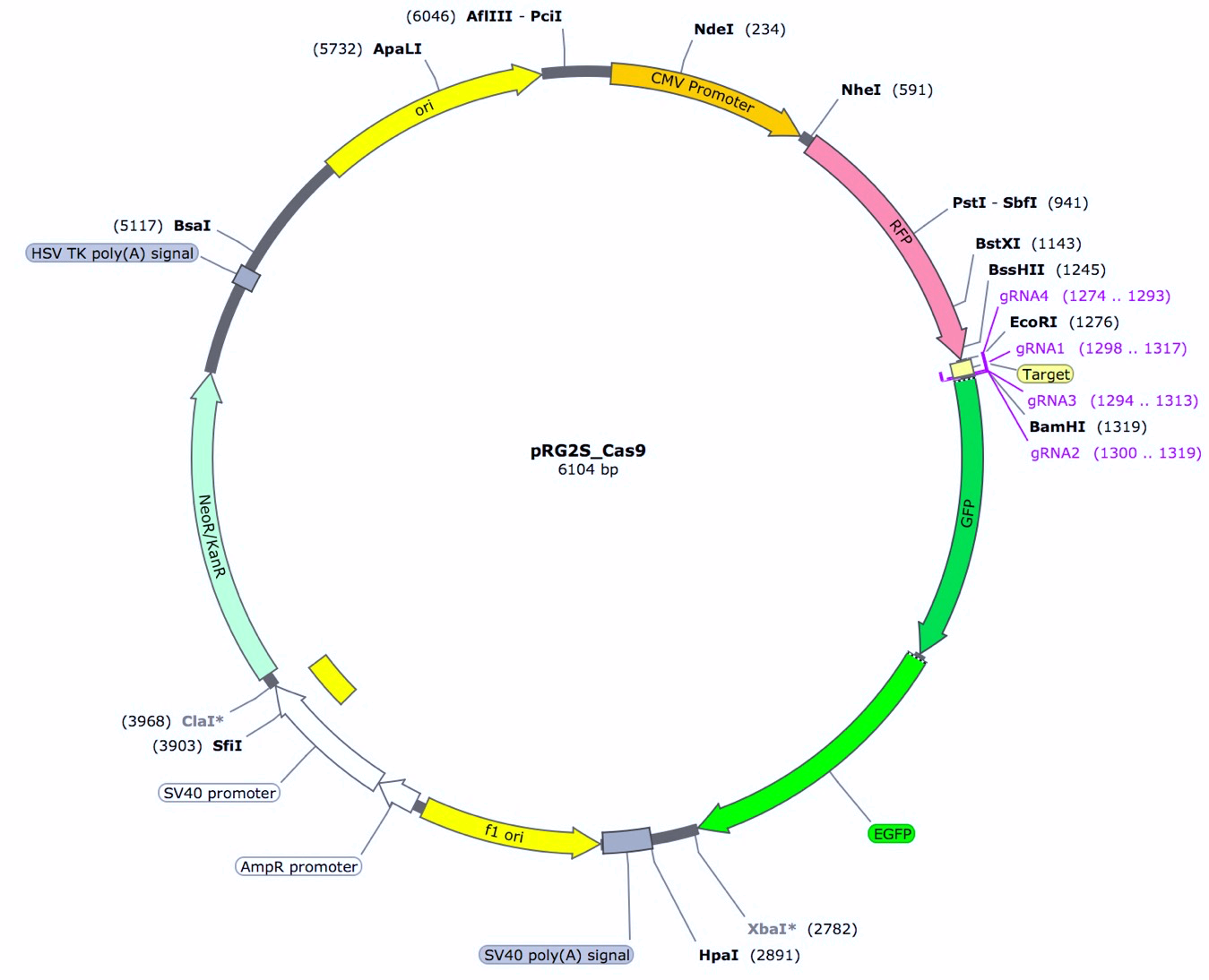


pRG2S_Cas9 reporter system including Cas9 target sequence, gRNA binding sites and restriction sites

**pX330-U6-Chimeric_BB-CBh-hSpCas9 (Cas9 expression plasmid) sequence and map:**

gagggcctatttcccatgattccttcatatttgcatatacgatacaaggctgttagagagataattggaattaatttgactgtaaacacaaagatattagtacaaaatacgtgacgtagaaagtaataatttcttgggtagtttgcagttttaaaattatgttttaaaatggactatcatatgcttaccgtaacttgaaagtatttcgatttcttggctttatatatcttgtggaaaggacgaaacaccgggtcttcgagaagacctgttttagagctagaaatagcaagttaaaataaggctagtccgttatcaacttgaaaaagtggcaccgagtcggtgcttttttgttttagagctagaaatagcaagttaaaataaggctagtccgtttttagcgcgtgcgccaattctgcagacaaatggctctagaggtacccgttacataacttacggtaaatggcccgcctggctgaccgcccaacgacccccgcccattgacgtcaatagtaacgccaatagggactttccattgacgtcaatgggtggagtatttacggtaaactgcccacttggcagtacatcaagtgtatcatatgccaagtacgccccctattgacgtcaatgacggtaaatggcccgcctggcattgtgcccagtacatgaccttatgggactttcctacttggcagtacatctacgtattagtcatcgctattaccatggtcgaggtgagccccacgttctgcttcactctccccatctcccccccctccccacccccaattttgtatttatttattttttaattattttgtgcagcgatgggggcggggggggggggggggsggggcggggggggggggggggsgrggsggrgrggkgcggcggcagccaatcagagcggcgcgctccgaaagtttccttttatggcgaggcggcggcggcggcggccctataaaaagcgaagcgcgcggcgggcgggagtcgctgcgcgctgccttcgccccgtgccccgctccgccgccgcctcgcgccgcccgccccggctctgactgaccgcgttactcccacaggtgagcgggcgggacggcccttctcctccgggctgtaattagctgagcaagaggtaagggtttaagggatggttggttggtggggtattaatgtttaattacctggagcacctgcctgaaatcactttttttcaggttggaccggtgccaccatggactataaggaccacgacggagactacaaggatcatgatattgattacaaagacgatgacgataagatggccccaaagaagaagcggaaggtcggtatccacggagtcccagcagccgacaagaagtacagcatcggcctggacatcggcaccaactctgtgggctgggccgtgatcaccgacgagtacaaggtgcccagcaagaaattcaaggtgctgggcaacaccgaccggcacagcatcaagaagaacctgatcggagccctgctgttcgacagcggcgaaacagccgaggccacccggctgaagagaaccgccagaagaagatacaccagacggaagaaccggatctgctatctgcaagagatcttcagcaacgagatggccaaggtggacgacagcttcttccacagactggaagagtccttcctggtggaagaggataagaagcacgagcggcaccccatcttcggcaacatcgtggacgaggtggcctaccacgagaagtaccccaccatctaccacctgagaaagaaactggtggacagcaccgacaaggccgacctgcggctgatctatctggccctggcccacatgatcaagttccggggccacttcctgatcgagggcgacctgaaccccgacaacagcgacgtggacaagctgttcatccagctggtgcagacctacaaccagctgttcgaggaaaaccccatcaacgccagcggcgtggacgccaaggccatcctgtctgccagactgagcaagagcagacggctggaaaatctgatcgcccagctgcccggcgagaagaagaatggcctgttcggaaacctgattgccctgagcctgggcctgacccccaacttcaagagcaacttcgacctggccgaggatgccaaactgcagctgagcaaggacacctacgacgacgacctggacaacctgctggcccagatcggcgaccagtacgccgacctgtttctggccgccaagaacctgtccgacgccatcctgctgagcgacatcctgagagtgaacaccgagatcaccaaggcccccctgagcgcctctatgatcaagagatacgacgagcaccaccaggacctgaccctgctgaaagctctcgtgcggcagcagctgcctgagaagtacaaagagattttcttcgaccagagcaagaacggctacgccggctacattgacggcggagccagccaggaagagttctacaagttcatcaagcccatcctggaaaagatggacggcaccgaggaactgctcgtgaagctgaacagagaggacctgctgcggaagcagcggaccttcgacaacggcagcatcccccaccagatccacctgggagagctgcacgccattctgcggcggcaggaagatttttacccattcctgaaggacaaccgggaaaagatcgagaagatcctgaccttccgcatcccctactacgtgggccctctggccaggggaaacagcagattcgcctggatgaccagaaagagcgaggaaaccatcaccccctggaacttcgaggaagtggtggacaagggcgcttccgcccagagcttcatcgagcggatgaccaacttcgataagaacctgcccaacgagaaggtgctgcccaagcacagcctgctgtacgagtacttcaccgtgtataacgagctgaccaaagtgaaatacgtgaccgagggaatgagaaagcccgccttcctgagcggcgagcagaaaaaggccatcgtggacctgctgttcaagaccaaccggaaagtgaccgtgaagcagctgaaagaggactacttcaagaaaatcgagtgcttcgactccgtggaaatctccggcgtggaagatcggttcaacgcctccctgggcacataccacgatctgctgaaaattatcaaggacaaggacttcctggacaatgaggaaaacgaggacattctggaagatatcgtgctgaccctgacactgtttgaggacagagagatgatcgaggaacggctgaaaacctatgcccacctgttcgacgacaaagtgatgaagcagctgaagcggcggagatacaccggctggggcaggctgagccggaagctgatcaacggcatccgggacaagcagtccggcaagacaatcctggatttcctgaagtccgacggcttcgccaacagaaacttcatgcagctgatccacgacgacagcctgacctttaaagaggacatccagaaagcccaggtgtccggccagggcgatagcctgcacgagcacattgccaatctggccggcagccccgccattaagaagggcatcctgcagacagtgaaggtggtggacgagctcgtgaaagtgatgggccggcacaagcccgagaacatcgtgatcgaaatggccagagagaaccagaccacccagaagggacagaagaacagccgcgagagaatgaagcggatcgaagagggcatcaaagagctgggcagccagatcctgaaagaacaccccgtggaaaacacccagctgcagaacgagaagctgtacctgtactacctgcagaatgggcgggatatgtacgtggaccaggaactggacatcaaccggctgtccgactacgatgtggaccatatcgtgcctcagagctttctgaaggacgactccatcgacaacaaggtgctgaccagaagcgacaagaaccggggcaagagcgacaacgtgccctccgaagaggtcgtgaagaagatgaagaactactggcggcagctgctgaacgccaagctgattacccagagaaagttcgacaatctgaccaaggccgagagaggcggcctgagcgaactggataaggccggcttcatcaagagacagctggtggaaacccggcagatcacaaagcacgtggcacagatcctggactcccggatgaacactaagtacgacgagaatgacaagctgatccgggaagtgaaagtgatcaccctgaagtccaagctggtgtccgatttccggaaggatttccagttttacaaagtgcgcgagatcaacaactaccaccacgcccacgacgcctacctgaacgccgtcgtgggaaccgccctgatcaaaaagtaccctaagctggaaagcgagttcgtgtacggcgactacaaggtgtacgacgtgcggaagatgatcgccaagagcgagcaggaaatcggcaaggctaccgccaagtacttcttctacagcaacatcatgaactttttcaagaccgagattaccctggccaacggcgagatccggaagcggcctctgatcgagacaaacggcgaaaccggggagatcgtgtgggataagggccgggattttgccaccgtgcggaaagtgctgagcatgccccaagtgaatatcgtgaaaaagaccgaggtgcagacaggcggcttcagcaaagagtctatcctgcccaagaggaacagcgataagctgatcgccagaaagaaggactgggaccctaagaagtacggcggcttcgacagccccaccgtggcctattctgtgctggtggtggccaaagtggaaaagggcaagtccaagaaactgaagagtgtgaaagagctgctggggatcaccatcatggaaagaagcagcttcgagaagaatcccatcgactttctggaagccaagggctacaaagaagtgaaaaaggacctgatcatcaagctgcctaagtactccctgttcgagctggaaaacggccggaagagaatgctggcctctgccggcgaactgcagaagggaaacgaactggccctgccctccaaatatgtgaacttcctgtacctggccagccactatgagaagctgaagggctcccccgaggataatgagcagaaacagctgtttgtggaacagcacaagcactacctggacgagatcatcgagcagatcagcgagttctccaagagagtgatcctggccgacgctaatctggacaaagtgctgtccgcctacaacaagcaccgggataagcccatcagagagcaggccgagaatatcatccacctgtttaccctgaccaatctgggagcccctgccgccttcaagtactttgacaccaccatcgaccggaagaggtacaccagcaccaaagaggtgctggacgccaccctgatccaccagagcatcaccggcctgtacgagacacggatcgacctgtctcagctgggaggcgacaaaaggccggcggccacgaaaaaggccggccaggcaaaaaagaaaaagtaagaattcctagagctcgctgatcagcctcgactgtgccttctagttgccagccatctgttgtttgcccctcccccgtgccttccttgaccctggaaggtgccactcccactgtcctttcctaataaaatgaggaaattgcatcgcattgtctgagtaggtgtcattctattctggggggtggggtggggcaggacagcaagggggaggattgggaagagaatagcaggcatgctggggagcggccgcaggaacccctagtgatggagttggccactccctctctgcgcgctcgctcgctcactgaggccgggcgaccaaaggtcgcccgacgcccgggctttgcccgggcggcctcagtgagcgagcgagcgcgcagctgcctgcaggggcgcctgatgcggtattttctccttacgcatctgtgcggtatttcacaccgcatacgtcaaagcaaccatagtacgcgccctgtagcggcgcattaagcgcggcgggtgtggtggttacgcgcagcgtgaccgctacacttgccagcgccttagcgcccgctcctttcgctttcttcccttcctttctcgccacgttcgccggctttccccgtcaagctctaaatcgggggctccctttagggttccgatttagtgctttacggcacctcgaccccaaaaaacttgatttgggtgatggttcacgtagtgggccatcgccctgatagacggtttttcgccctttgacgttggagtccacgttctttaatagtggactcttgttccaaactggaacaacactcaactctatctcgggctattcttttgatttataagggattttgccgatttcggtctattggttaaaaaatgagctgatttaacaaaaatttaacgcgaattttaacaaaatattaacgtttacaattttatggtgcactctcagtacaatctgctctgatgccgcatagttaagccagccccgacacccgccaacacccgctgacgcgccctgacgggcttgtctgctcccggcatccgcttacagacaagctgtgaccgtctccgggagctgcatgtgtcagaggttttcaccgtcatcaccgaaacgcgcgagacgaaagggcctcgtgatacgcctatttttataggttaatgtcatgataataatggtttcttagacgtcaggtggcacttttcggggaaatgtgcgcggaacccctatttgtttatttttctaaatacattcaaatatgtatccgctcatgagacaataaccctgataaatgcttcaataatattgaaaaaggaagagtatgagtattcaacatttccgtgtcgcccttattcccttttttgcggcattttgccttcctgtttttgctcacccagaaacgctggtgaaagtaaaagatgctgaagatcagttgggtgcacgagtgggttacatcgaactggatctcaacagcggtaagatccttgagagttttcgccccgaagaacgttttccaatgatgagcacttttaaagttctgctatgtggcgcggtattatcccgtattgacgccgggcaagagcaactcggtcgccgcatacactattctcagaatgacttggttgagtactcaccagtcacagaaaagcatcttacggatggcatgacagtaagagaattatgcagtgctgccataaccatgagtgataacactgcggccaacttacttctgacaacgatcggaggaccgaaggagctaaccgcttttttgcacaacatgggggatcatgtaactcgccttgatcgttgggaaccggagctgaatgaagccataccaaacgacgagcgtgacaccacgatgcctgtagcaatggcaacaacgttgcgcaaactattaactggcgaactacttactctagcttcccggcaacaattaatagactggatggaggcggataaagttgcaggaccacttctgcgctcggcccttccggctggctggtttattgctgataaatctggagccggtgagcgtggaagccgcggtatcattgcagcactggggccagatggtaagccctcccgtatcgtagttatctacacgacggggagtcaggcaactatggatgaacgaaatagacagatcgctgagataggtgcctcactgattaagcattggtaactgtcagaccaagtttactcatatatactttagattgatttaaaacttcatttttaatttaaaaggatctaggtgaagatcctttttgataatctcatgaccaaaatcccttaacgtgagttttcgttccactgagcgtcagaccccgtagaaaagatcaaaggatcttcttgagatcctttttttctgcgcgtaatctgctgcttgcaaacaaaaaaaccaccgctaccagcggtggtttgtttgccggatcaagagctaccaactctttttccgaaggtaactggcttcagcagagcgcagataccaaatactgttcttctagtgtagccgtagttaggccaccacttcaagaactctgtagcaccgcctacatacctcgctctgctaatcctgttaccagtggctgctgccagtggcgataagtcgtgtcttaccgggttggactcaagacgatagttaccggataaggcgcagcggtcgggctgaacggggggttcgtgcacacagcccagcttggagcgaacgacctacaccgaactgagatacctacagcgtgagctatgagaaagcgccacgcttcccgaagggagaaaggcggacaggtatccggtaagcggcagggtcggaacaggagagcgcacgagggagcttccagggggaaacgcctggtatctttatagtcctgtcgggtttcgccacctctgacttgagcgtcgatttttgtgatgctcgtcaggggggcggagcctatggaaaaacgccagcaacgcggcctttttacggttcctggccttttgctggccttttgctcacatgt

**
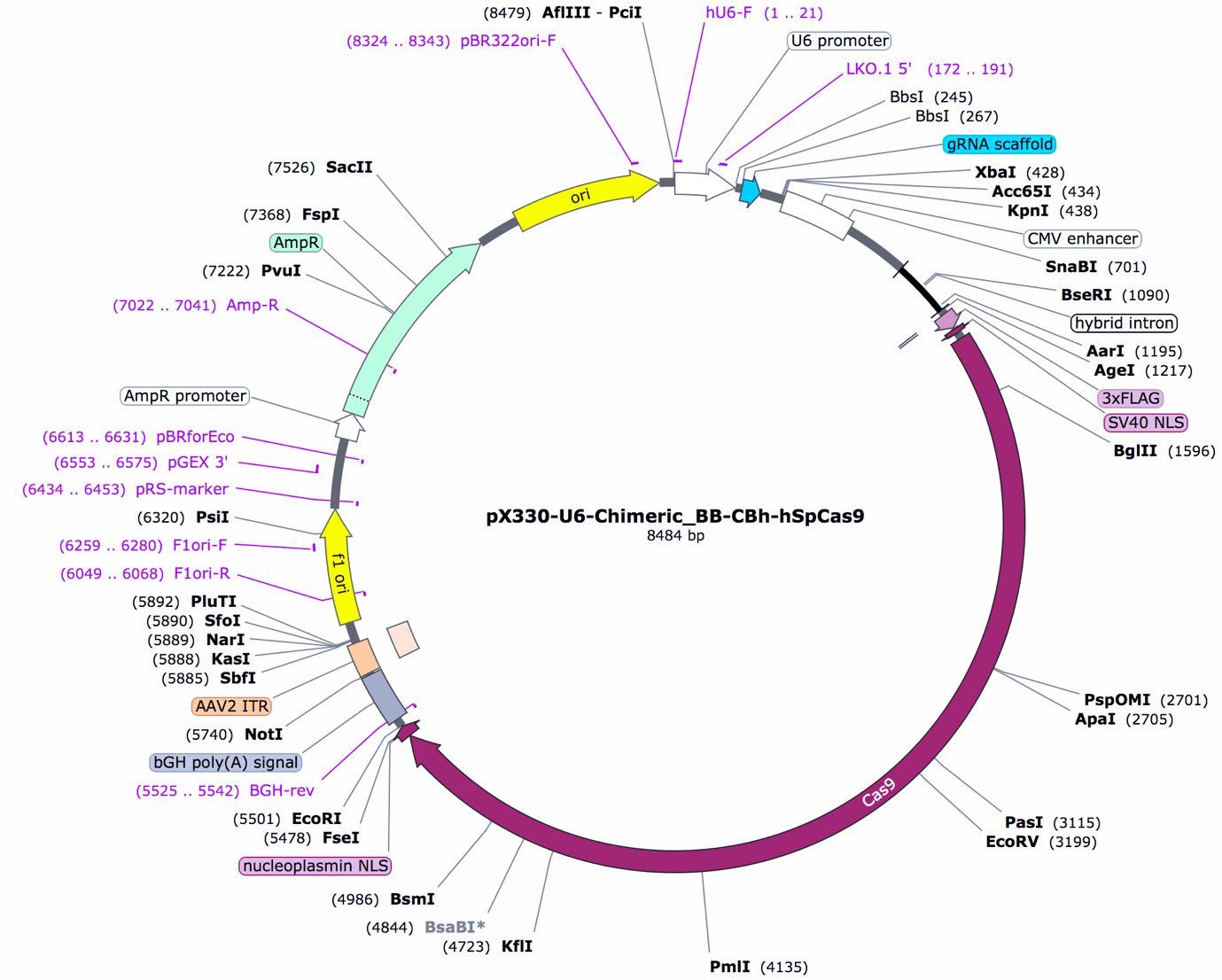
**

pX330-U6-Chimeric_BB-CBh-hSpCas9 Cas9 expression plasmid with gRNA scaffold and gRNA between BbsI restriction sites

**Sanger Sequences of untransfected (wildtype) and transfected cells used for TIDE analysis:**

**pCas9/gSYNE4 plasmid-treated HEK293 cells (SYNE4_Reverse primer):**

**Untransfected:**

GRCGSRKRRYYTKGTYCAGGGTCTGGGCCTMSTCTGGGCTAGGAGGCAGGGGGCGGTGACTGGGTGAGTCTCGMCAYYTCACTTTTCAGCCTACCGTCACCCCAACCCTGGTGGCCTCTCCTATGTCCCCAGGCGATCACAGCCATGGCACCTCCTACCCTCACATGGCCCCTGCCACCACGCCTACCCTTTTGGGAACAAGCTTCCAAAGCCCCGGCCCCCACCTCGTGCTCTCCTCTCCGGACGCGGGGCAGACGGTGCATCCAACAATGTCCGCCTCTCTAGGTGCTCCCGGTGGGTGGTTGAGGGGCTCTGAGCCAAGTCTAGGGCCCAGAGGCAGGGACAGGGCCATGGCTGGGGGCCTGGGGACACAAAGTCAGGTGAGGGCAGCCAGTAAGACCTCTTCCCTAGACAAGGGTGTCCCAGAGCTCCTCCGCTGGAGTCACCCGGGCCTGAGGCTGCAGRA

**RFP^+^GFP^+^:**

GGGARRRWCKGTWCAGGGGTCTGGGGCTGCTCTGGGCTAGGAGGCAGGGGGCGGTGACTGGGTGAGTCTCGACACCTCACTTTTCAGCCTACCGTCACCCCAACCCTGGTGGCCTCTCCTATGTCCCCAGGCGATCACAGCCATGGCACCTCCTACCCTCACATGGCCCCTGCCACCACGCCTACCCTTTTGGGAACAAGCTTCCAAAGCCCCGGCCCCCACCTCGTGCTCTCCTCTCCGGACGCGGGGCAGACGGTGCATCCAACAATGTCCGCCTCTCTAGGTGCTCCCGGTGGGTGGTTGAGGGGCTCTGAGCCAAGTCTAGGGCCCAGAGMSAGGCACGGGGCCCGGGGTGGGGGCACGGGGACAAGRGAGAGGGGAGGGCCAGYCACCTAACCTCTATACTAGAGAAAGGGAGACCCCAACCTCCCCCGTGGCRGGCCCCKGGGAWTGGAGATKMRAAA

**pCas9/gEMX1 plasmid-treated HEK293 cells (EMX1_Forward primer):**

**Untransfected:**

TGGRCKCAGKGCGGGCGGGCCGCCCAGGCAGGCAGGCTCTCCGAGGAGAAGGCCAAGTGGTCCCAGGCCTCAGCCAGCCCATTGCTTGTCCCTCTGTCAATGGCGGCCCCGGGCTTCAAGCCCTGTGGGGCCATGACTCCAGGCCTCCCCAAAGCCTGGCCAGGGAGTGGCCAGAGTCCAGCTTGGGCCCACGCAGGGGCCTGGCCAGCAGCAAGCAGCACTCTGCCCTCGTGGGTTTGTGGTTGCCCACCCTAGTCATTGGAGGTGACATCGATGTCCTCCCCATTGGCCTGCTTCGTGGCAATGCGCCACCGGTTGATGTGATGGGAGCCCTTCTTCTTCTGCTCGGACTCAGGCCCTTCCTCCTCCAGCTTCTGCCGTTTGTACTTTGTCCTCCGGTTCTGGAACCACACCTTCACCTGGGCCAGGGAGGGAGGGGCACAGATGAGAAACTCAGGAGGCCCCCAGAGCAGCCACTGGGGCCTCAACACTCAGGCTGAGCTGAGAGCCTGATGGGAAGACTGAGGCTACATAGGGTTAGGGGCCCCAGGCCGGGGTCCCCTCTGACCAGCTGCTCCCATGGGTCTAACATTCAARAARGGGGRRGAGGG

**RFP^+^GFP^+^:**

TGGRCKCAGKGCGGGCGGGCCGCCCAGGCAGGCAGGCTCTCCGAGGAGAAGGCCAAGTGGTCCCAGGCCTCAGCCAGCCCATTGCTTGTCCCTCTGTCAATGGCGGCCCCGGGCTTCAAGCCCTGTGGGGCCATGACTCCAGGCCTCCCCAAAGCCTGGCCAGGGAGTGGCCAGAGTCCAGCTTGGGCCCACGCAGGGGCCTGGCCAGCAGCAAGCAGCACTCTGCCCTCGTGGGTTTGTGGTTGCCCACCCTAGTCATTGGAGGTGACATCGATGTCCTCCCCATTGGCCTGCTTCGTGGCAATGCGCCACCGGTTGATGTGATGGGAGCCCTTCTTCTTCTGCTCGGACTCAGGCCCTTCCTCCTCCAGCTTCTGCCGTTTGTACTTTGTCCTCCGGTTCTGGAACCACACCTTCACCTGGGCCAGGGAGGGAGGGGCACAGATGAGAAACTCAGGAGGCCCCCAGAGCAGCCACTGGGGCCTCAACACTCAGGCTGAGCTGAGAGCCTGATGGGAAGACTGAGGCTACATAGGGTTAGGGGCCCCAGGCCGGGGTCCCCTCTGACCAGCTGCTCCCATGGGTCTAACATTCAARAARGGGGRRGAGGG

**Cas9/gSNCA4 RNP-treated HEK293 cells (SNCA_Forward primer):**

**Untransfected:**

GGTTCCGGCGAATTGTTTACTGCATGACTTTAGCCAAATRTCTTAGCCAAGATTCAATGTTTGGTTGAACCACACTCACTTGGACATCTTGGTGGCTTTTGTTTCTTCTGACCACTCAGTTATCTATGGCATGTGTAGATACAGGTGTATGGAAGCCGATGGCTAGTGGAAGTGGAATGATTTTAAGTCACTGTTATTCTACCACCCTTTAATCTGTTGTTGCTCTTTATTTGTACCAGTGGCTGAGAAGACCAAAGAGCAAGTGACAAATGTTGGAGGAGCAGTGGTGACGGGTGTGACAGCAGTAGCCCAGAAGACAGTGGAGGGAGCAGGGAGCATTGCAGCAGCCACTGGCTTTGTCAAAAAGGACCAGTTGGGCAAGGTATGGCTGTGTACGTTTTGTGTTACATTTATAAGCTGGTGAGATTACGGTTCATTTTCATGTGAGGCCTGGAGGCAGGAGCAAGATACTTACTGTGGGGAACGGCTACCTGAAAGAA

**RFP^+^GFP^+^:**

GTTTCGGCACTGTTTACTGCATGACTTTAGCCAATRTCTTAGCCAAGATTCAATGTTTGGTTGAACCACACTCACTTGGACATCTTGGTGGCTTTTGTTTCTTCTGACCACTCAGTTATCTATGGCATGTGTAGATACAGGTGTATGGAAGCCGATGGCTAGTGGAAGTGGAATGATTTTAAGTCACTGTTATTCTACCACCCTTTAATCTGTTGTTGCTCTTTATTTGTACCAGTGGCTGAGAAGACCAAAGAGCAAGTGACAAAWKKTKGRRGRRSMRKGGKGAMSGGKGGGACCRCCRTARCCCAAAAAAAAGGGGAAGGAGCAGGGAGCATTGCMRCCRCCACTGGSTTTGGCAAAAAAGAACARKTGGGSMAGGGWTGGSTGGGTWCSTTTTGGGTTACATTTATTAACTGGGGAGATTACCGGTCATTTTCATGGGAGGGCTGGAAGGMRGARSMAGAAACTTACTGGGGGGAAACGSYACCTGGAAGAAA

**Cas9/gSNCA4 plasmid-treated SH-SY5Y cells (SNCA_Forward primer):**

**Untransfected:**

GTTTGGGCTCTGTTTACTGCATGACTTTAGCCAATATCTTAGCCAAGATTCAATGTTTGGTTGAACCACACTCACTTGGACATCTTGGTGGCTTTTGTTTCTTCTGACCACTCAGTTATCTATGGCATGTGTAGATACAGGTGTATGGAAGCCGATGGCTAGTGGAAGTGGAATGATTTTAAGTCACTGTTATTCTACCACCCTTTAATCTGTTGTTGCTCTTTATTTGTACCAGTGGCTGAGAAGACCAAAGAGCAAGTGACAAATGTTGGAGGAGCAGTGGTGACGGGTGTGACAGCAGTAGCCCAGAAGACAGTGGAGGGAGCAGGGAGCATTGCAGCAGCCACTGGCTTTGTCAAAAAGGACCAGTTGGGCAAGGTATGGCTGTGTACGTTTTGTGTTACATTTATAAGCTGGTGAGATTACGGTTCATTTTCATGTGARGCCTGGAGGCAGGRRSMARRWWMYTWMTGGGGGGRAASGSYWCCCTASAKRAA

**RFP^+^GFP^+^:**

GGTTCAGCATAGTTTGTACTGCATGACTTTAGCCAAAATATCTTAGCCAAGATTCAATGTTTGGTTGAACCACACTCACTTGGACATCTTGGTGGCTTTTGTTTCTTCTGACCACTCAGTTATCTATGGCATGTGTAGATACAGGTGTATGGAAGCCGATGGCTAGTGGAAGTGGAATGATTTTAAGTCACTGTTATTCTACCACCCTTTAATCTGTTGTTGCTCTTTATTTGTACCAGTGGCTGAGAAGACCAAAGAGCAAGTGACAAATGTTGGARGARSAGKGGTGACGGGTGTGACCSCMGKASCCCAAAAAACGGGGGAGGGAGGGGGGAGCWTTGCMSCMSCCMCTGGYTTTKKMAAAAAGGACCAGTTGGGSAAGGTATGGYTGTGTTCTTTTTGTGTTACATTTATAAGSTGGKGAGATTWCSGTTYATTTTCMTGTGAAGSCTGGAGGGRGGGASMAAAATMTTTYCGGGGGGRAAGSCYMMMMMWAAAAAAA
